# Soybean anthracnose caused by *Colletotrichum* species: Current status and future prospects

**DOI:** 10.1111/mpp.13036

**Published:** 2021-02-20

**Authors:** Thais R. Boufleur, Maisa Ciampi‐Guillardi, Ísis Tikami, Flávia Rogério, Michael R. Thon, Serenella A. Sukno, Nelson S. Massola Júnior, Riccardo Baroncelli

**Affiliations:** ^1^ Department of Plant Pathology and Nematology University of São Paulo (USP), Luiz de Queiroz College of Agriculture (ESALQ) Piracicaba, São Paulo Brazil; ^2^ Instituto Hispano‐Luso de Investigaciones Agrarias (CIALE) Universidad de Salamanca Salamanca Spain

**Keywords:** *Colletotrichum truncatum*, emerging diseases, fungal pathogens, *Glomerella*, *Glycine max*

## Abstract

Soybean (*Glycine max*) is one of the most important cultivated plants worldwide as a source of protein‐rich foods and animal feeds. Anthracnose, caused by different lineages of the hemibiotrophic fungus *Colletotrichum*, is one of the main limiting factors to soybean production. Losses due to anthracnose have been neglected, but their impact may threaten up to 50% of the grain production.

**Taxonomy:**

While *C. truncatum* is considered the main species associated with soybean anthracnose, recently other species have been reported as pathogenic on this host. Until now, it has not been clear whether the association of new *Colletotrichum* species with the disease is related to emerging species or whether it is due to the undergoing changes in the taxonomy of the genus.

**Disease symptoms:**

Typical anthracnose symptoms are pre‐ and postemergence damping‐off; dark, depressed, and irregular spots on cotyledons, stems, petioles, and pods; and necrotic laminar veins on leaves that can result in premature defoliation. Symptoms may evolve to pod rot, immature opening of pods, and premature germination of grains.

**Challenges:**

As accurate species identification of the causal agent is decisive for disease control and prevention, in this work we review the taxonomic designation of *Colletotrichum* isolated from soybean to understand which lineages are pathogenic on this host. We also present a comprehensive literature review of soybean anthracnose, focusing on distribution, symptomatology, epidemiology, disease management, identification, and diagnosis. We consider the knowledge emerging from population studies and comparative genomics of *Colletotrichum* spp. associated with soybean providing future perspectives in the identification of molecular factors involved in the pathogenicity process.

**Useful website:**

Updates on *Colletotrichum* can be found at http://www.colletotrichum.org/.

All available *Colletotrichum* genomes on GenBank can be viewed at http://www.colletotrichum.org/genomics/.

## Graphical abstract

A revision of soybean anthracnose revealed that along with *Colletotrichum truncatum*, several other species of *Colletotrichum* are associated with soybean worldwide, revealing a knowledge gap in what we know about this disease so far.

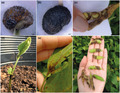

## 1 INTRODUCTION

The genus *Colletotrichum* constitutes a large monophyletic group of ascomycetes with more than 200 accepted species, classified into at least 14 species complexes (s.c.) and singletons (Damm et al., 2019; Marin‐Felix et al., 2017). Considered as among the top 10 plant pathogenic fungi, *Colletotrichum* spp. are the causal agents of anthracnose in more than 3,000 plant species, leading to considerable yield reduction of economically important crops (Cannon et al., 2012; Dean et al., 2012; da Silva et al., 2020). Due to its hemibiotrophic lifestyle and the facility of being manipulated in the laboratory, the genus is considered a model pathogen for biochemical, physiological, and genetic studies (Baroncelli et al., 2017; O’Connell et al., 2012; Perfect et al., 1999).

The soybean crop has great importance worldwide as a source of vegetable oil and proteins for human and animal feeds (Hartman et al., 2011; Pagano & Miransari, 2016), contributing 3.3% of the global human calorie intake (FAOSTAT, 2018). In 2019/20, world soybean production exceeded 330 million tonnes, of which approximately 86% were concentrated in Brazil, the United States, and Argentina (USDA, 2020). Diseases are among the major factors that can affect soybean production, and anthracnose is becoming a major threat in production areas around the world (Dias et al., 2016; Hartman et al., 2015; Nataraj et al., 2020; Subedi et al., 2016; Wrather et al., 2010;). This disease can reach up to 100% incidence in the field (Hartman et al., 1999), and incidence as low as 1% can cause yield losses of up to 90 kg/ha (Dias et al., 2016).

Soybean anthracnose is currently recognized as a disease of complex aetiology (Dias et al., 2018), with *C. truncatum* the most common associated species (Sharma et al., 2011). In recent years, several other species have been reported as causal agents of the disease, such as *C. destructivum* (Manandhar et al., 1986), *C. coccodes* (Riccioni et al., 1998), *C. chlorophyti* (Yang et al., 2012, 2013), *C. gloeosporioides* (Mahmodi et al., 2013), *C. incanum* (Yang et al., 2014), *C. plurivorum* (Barbieri et al., 2017), *C. sojae* (Damm et al., 2019), and, more recently, *C. musicola* (Boufleur et al., 2020) and *C. brevisporum* (Shi et al., 2020).

*Colletotrichum* species can affect soybean in all physiological stages (Sharma et al., 2011). Typical symptoms of anthracnose are pre‐ and postemergence damping‐off; and dark, depressed, and irregular spots on stems, petioles, and pods that can evolve and cause premature defoliation of the plants (Yang et al., 2015). Such infections have the potential to cause severe losses that can reach up to 100% in soybean‐producing areas under favourable conditions (Yang & Hartman, 2016).

An accurate species identification of a causal agent is decisive for disease control and prevention. The occurrence of multiple *Colletotrichum* species associated with soybean anthracnose may affect disease management because distinct species might respond differently to different control strategies. Different studies about the efficiency of fungicides in the control of soybean anthracnose showed contradictory results (Chen et al., 2018; Dias et al., 2016; Poti et al., 2020), which could be due to different responses of *Colletotrichum* species to their active compounds.

Few discriminatory morphological characters are available, and the identification of *Colletotrichum* species based exclusively on these features is unreliable (Cai et al., 2009; Jayawardena et al., 2016). Currently, species identification of this genus is performed using a polyphasic approach that combines morphological and cultural characteristics with multilocus phylogenetic analyses of DNA sequences (Cai et al., 2009; Liu et al., 2016).

Several species within the *Colletotrichum* genus show a wide genetic variability; however, the mechanisms responsible for such diversity are not yet fully understood (da Silva et al., 2020). Some population genetics studies of *Colletotrichum* species have offered tools for improving prevention and management strategies for plant diseases of important agricultural crops (Baroncelli et al., 2015; Ciampi‐Guillardi et al., 2014; Rogério et al., 2019; Ureña‐Padilla et al., 2002).

Many unresolved questions about soybean anthracnose remain. Until now, it has not been clear whether the association of new *Colletotrichum* species with the disease is related to emerging species or whether it is due to the ongoing changes in the taxonomy of the genus. Most of the studies available for soybean anthracnose are focused on *C. truncatum*, with limited information about the other *Colletotrichum* species infecting this crop, which could result in obstacles for the management of the disease in the field. The aim of this work is to gain a better understanding of soybean anthracnose and its causal agents, clarify which *Colletotrichum* species or complexes are associated with the disease, and provide a comprehensive review for future studies on soybean anthracnose.

## 2 *COLLETOTRICHUM* SPECIES ASSOCIATED WITH SOYBEAN, LIFESTYLE, AND GEOGRAPHIC DISTRIBUTION

The lack of reliable morphological characteristics has made the identification of *Colletotrichum* spp. a major challenge over the years (Cai et al., 2009), leading to considerable taxonomic confusion. After the advent of DNA‐based characterization, the taxonomy and nomenclature of *Colletotrichum* spp. underwent many revisions (Cannon et al., 2012; Damm et al., 2009, 2012a,2012b, 2014, 2019; Jayawardena et al., 2016; Liu et al., 2014; Marin‐Felix et al., 2017; Weir et al., 2012). Currently, there are more than 200 recognized *Colletotrichum* species, either as singletons or as part of 14 s.c. (Damm et al., 2019; Marin‐Felix et al., 2017). Classification of *Colletotrichum* into s.c. can be done using the internal transcribed spacer (ITS)‐5.8S rRNA region. A correct species identification requires a multilocus approach, with distinct s.c. demanding different loci to be analysed (Damm et al., 2019; Marin‐Felix et al., 2017).

Soybean anthracnose was first described in Korea in 1917, associated with *C. glycines* (Nakata & Takimoto, 1934). The same species was also reported as pathogenic to soybean in the USA in 1926 (Lehman & Wolf, 1926). Later, *C. glycines* (a synonym of *C. truncatum*) and *Glomerella glycines* were reported on soybean (Damm et al., 2019; Sharma et al., 2011; Sinclair, 1989). Until recently, studies of this disease have been mainly focused on *C. truncatum*. In contrast, the *C. orchidearum* s.c. was described only recently (Damm et al., 2019), and three species within this complex have already been reported as pathogenic to soybean (Barbieri et al., 2017; Boufleur et al., 2020; Lehman & Wolf, 1926). The recent massive taxonomic revision of this genus has led to an increase of taxonomically wrongly assigned ITS sequences deposited in GenBank (Crouch, Clarke, et al., 2009; Damm et al., 2009). To clarify the *Colletotrichum* s.c. associated with soybean anthracnose, and the worldwide distribution of this disease, all publicly available ITS sequences of *Colletotrichum* isolated from soybean and relative information were retrieved and compared with those of reference isolates through a phylogenetic analysis.

**FIGURE 1:**
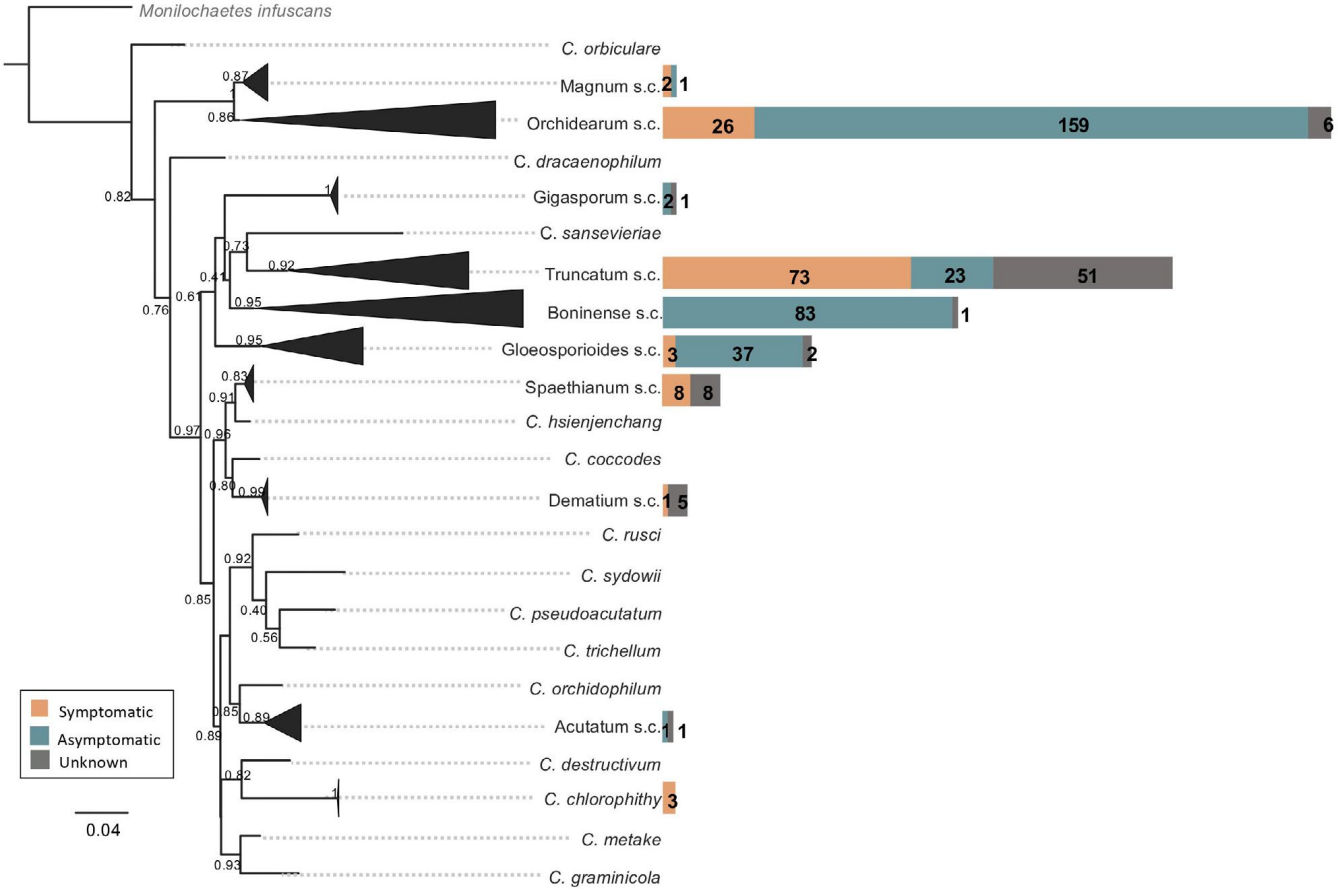
*Colletotrichum* species complexes (s.c.) associated with soybean worldwide identified based on a Bayesian phylogenetic analysis of internal transcribed spacer (ITS) sequences. *Colletotrichum* s.c. associated with symptomatic, asymptomatic, and unknown soybean plants are indicated by bars of distinct colours. All sequences were aligned using MAFFT v. 7.450 (Katoh, 2002; Katoh & Standley, 2013) and the multiple sequence alignment was exported to MEGA 10 (Stecher et al., 2020), in which the best‐fit substitution model was calculated for the sequence data set. The concatenated alignment was performed with Geneious v. 2020.0.4 (https://www.geneious.com). A Markov chain Monte Carlo (MCMC) algorithm was used to generate phylogenetic trees with Bayesian probabilities with MrBayes v. 3.2.6 (Huelsenbeck & Ronquist, 2001) based on the model of nucleotide substitution. The analyses were run from random trees for 5,000,000 generations and sampled every 1,000 generations. The concatenated tree was compressed in FigTree v. 1.4.4 (Rambaut, 2014)

The in silico screening for *Colletotrichum* species isolated from soybean plants with and without symptoms whose nucleotide sequences are deposited in GenBank yielded 499 ITS sequences originally assigned to eight *Colletotrichum* s.c. (*C. acutatum*, *C. boninense*, *C*. *dematium*, *C*. *gloeosporioides*, *C. magnum*, *C*. *orchidearum*, *C. spaethianum*, and *C. truncatum*) and one singleton species (*C. chlorophyti*) (Table S1). While it has been shown that around 10% of all deposited ITS sequences are assigned to the wrong species (Nilsson et al., 2006), this number seems to be much higher in the case of *Colletotrichum*. In our data set more than 37% of the sequences were wrongly assigned at the s.c. level and therefore the proportion could be higher at the species level (Table S1). Our analyses suggest that *Colletotrichum* strains isolated from soybean belong to nine s.c. (*C. acutatum*, *C*. *boninense*, *C. dematium*, *C. gloeosporioides*, *C. gigasporum*, *C. magnum*, *C*. *orchidearum*, *C. spaethianum*, and *C. truncatum*) and one singleton species (*C. chlorophyti*) (Figure 1). To our knowledge this is the first time that species belonging to the *C. gigasporum* s.c. have been associated with soybean (Table 1).

**TABLE 1:** *Colletotrichum* species complexes associated with soybean anthracnose

Species complex	Species	Distribution	Lifestyle	References
*C. acutatum*	Unknown	Brazil, USA	Endophyte	Leite et al., 2013
*C. boninense*	Unknown^1^	Brazil, Taiwan	Endophyte	Leite et al., 2013
*C. gloeosporioides*	*C. gloeosporioides, C. salsolae*	Brazil, Colombia, Hungary, Taiwan	Pathogen, endophyte	Weir et al., 2012; Leite et al., 2013; Chen et al., 2006
*C. spaethianum*	*C. incanum*	Canada, USA	Pathogen	Yang et al., 2014
*C. gigasporum*	Unknown^1^	Brazil	Endophyte	Leite et al., 2013
*C. dematium*	Unknown^1^	Canada	Pathogen	Unpublished
None	*C. chlorophyti*	USA	Pathogen	Cannon et al., 2012; Yang et al., 2012; Yang et al., 2014
*C. magnum*	*C. brevisporum*	Brazil, China, Taiwan	Pathogen, endophyte	Leite et al., 2013; Shi et al., 2020
*C. orchidearum*	*C. musicola, C. plurivorum, C. sojae*	Brazil, Iran, Italy, Japan, Malasya, Myanmar, Serbia, Taiwan, USA	Pathogen, endophyte	Riccioni et al., 1998; Leite et al., 2013; Barbieri et al., 2017; Batzer & Muller, 2020 ; Damm et al., 2019; Boufleur et al., 2020; Zaw & Aye, 2020
*C. truncatum*	*C. truncatum*	Brazil, Canada, China, Colombia, South Korea, Taiwan, USA	Pathogen, endophyte	Chen et al., 2006; Damm et al., 2009; Leite et al., 2013; Yang et al., 2014; Rogério et al., 2017; Zaw & Aye, 2020

^1^ No species could be assigned to the correct taxonomic position within the species complex due the lack of available information.

Although several s.c. have been associated with soybean (Figure 1), it was not possible to confirm if the strains belonging to the *C. acutatum*, *C. boninense*, and *C. gigasporum* s.c. are truly pathogenic to soybean, as all the sequences retrieved that belong to those complexes came from symptomless plants or information related to pathogenicity was not available. It is known that *Colletotrichum* can go through a quiescent phase before the development of disease symptoms on the host (Prusky, 1996; Prusky et al., 2013; de Silva et al., 2017) and can live inside nonhost plant tissues as endophytes (da Silva et al., 2020). Strains belonging to the *C. dematium*, *C. magnum*, *C. gloeosporioides*, *C. orchidearum*, *C. truncatum* s.c., and *C. chlorophyti* have been confirmed to be pathogenic (Table 1).

*C. coccodes* and *C. destructivum* were also reported to be pathogenic to soybean (Manandhar et al., 1986; Riccioni et al., 1998), but no genetic information is available and therefore we could not confirm the taxonomic designation of the strains used in those studies. *C. coccodes* is a singleton species isolated from soybean in the USA (Riccioni et al., 1998) and *C. destructivum* was reported for the first time as pathogenic to soybean in the 1980s, associated with the sexual morph *G. glycines* (Manandhar et al., 1986); thus, this species was recently reclassified as *C. sojae* (Damm et al., 2019). One of the most important soybean diseases in Argentina, the third major soybean producer of the world (USDA, 2020), is the late season disease complex that includes soybean anthracnose caused by *C. truncatum* and *C. destructivum* (Ramos et al., 2010, 2013). However, the real association of *C. destructivum* with the disease remains unclear, because those species were identified based only on morphological characteristics (Ramos et al., 2013).

**FIGURE 2:**
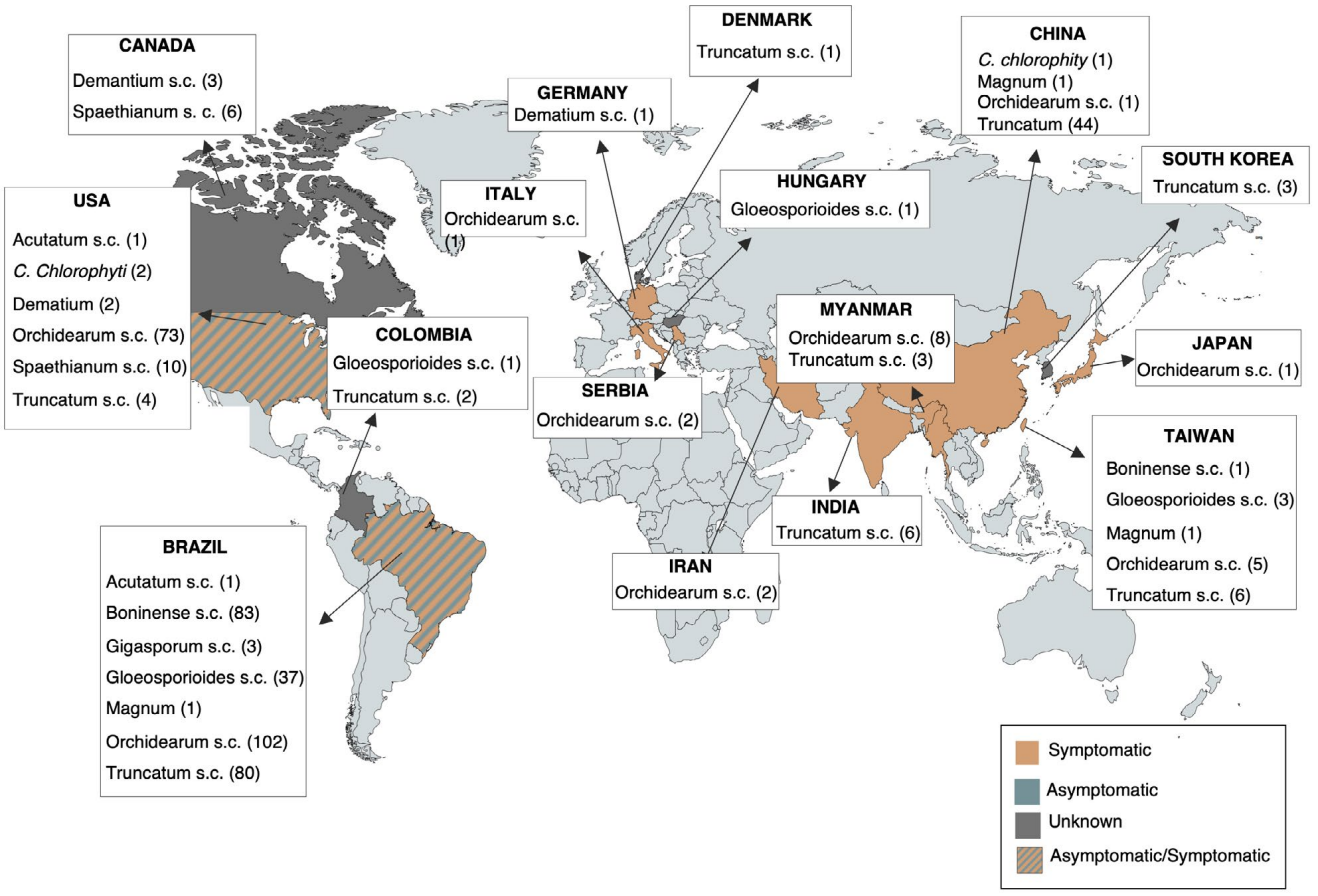
Global distribution of the *Colletotrichum* species complexes (s.c.) associated with soybean based on the information downloaded with sequences from GenBank. The number of isolates in each country or region is presented to the side of the names of the respective species complex. Countries or regions with the presence of symptomatic, asymptomatic, and unknown soybean plants are indicated by different colours

Sixteen countries or regions around the world reported the presence of at least one *Colletotrichum* lineage associated with soybean. Brazil, the USA, and Taiwan have the largest diversity, followed by China, Colombia, Canada, and Myanmar (Figure 2). *C. orchidearum* and *C. truncatum* s.c. were the most widely distributed worldwide, present in nine countries, with the largest number of ITS sequences retrieved followed by the *C. gloeosporioides* s.c. present in four different countries (Figures 1 and 2), whereas the other s.c. seem to be restricted to one or two countries (Figure 2). Considering the number of records and the distribution of the *C. orchidearum* s.c. strains isolated since 2003 (Table S1) and their presence in several soybean‐producing countries, this complex is probably more important in the epidemiology of the disease than is currently assumed; thus, more studies need to be performed to understand its distribution, host specificity, and impact in soybean fields around the world.

How many and which *Colletotrichum* species can be pathogenic or endophytic to soybean is still unclear. Historically, *C. truncatum* has been considered the prevalent species isolated from soybean or associated with soybean anthracnose (Sharma et al., 2011). However, many questions remain open, and based on the genetic data available (Table S1) we can hypothesize that the importance of *C. truncatum* has been overestimated. Precise knowledge about pathogen taxonomic designation and diversity is crucial, having direct implications on disease management, either by cultural or chemical strategies, as well as on disease resistance breeding programmes (Chen et al., 2018).

## 3 SYMPTOMATOLOGY AND EPIDEMIOLOGY

**FIGURE 3:**
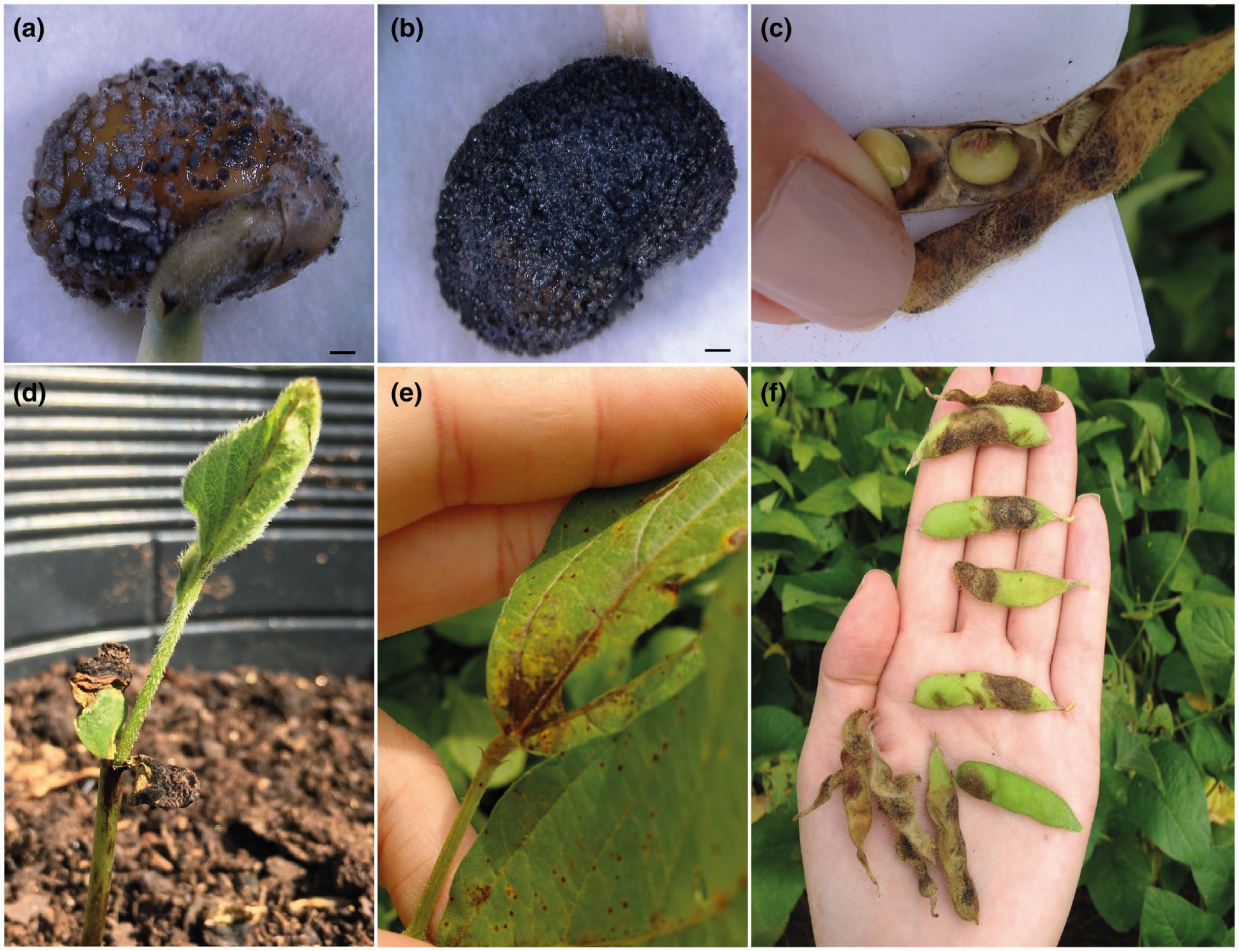
Typical symptoms and signs of *Colletotrichum truncatum* on infected soybean seeds (a–c). Dark, depressed, and irregular spots on cotyledons, stem, petioles, and pods (d–f)

Favoured by warm and humid conditions, typical anthracnose symptoms can appear on all parts of soybean plants and in all physiological stages (Yang & Hartman, 2016). Systemic infections on seeds can cause pre‐ and/or postemergence damping‐off and cotyledon lesions (Sharma et al., 2011). Symptoms are commonly characterized by dark, depressed, and irregular spots on stems, petioles, and pods. Leaves may be shrunken, rolled or wilted, and may have necrotic laminar veins, resulting in premature defoliation of the plants (Figure 3) (Yang et al., 2015). This pattern of symptoms is reported for *C. truncatum*, *C. coccodes*, *C. gloeosporioides*, *C. plurivorum*, *C. musicola*, and *C. incanum* (Boufleur et al., 2020; Dias et al., 2018; Mahmodi et al., 2013; Riccioni et al., 1998; Sharma et al., 2011; Yang et al., 2014), while *C. sojae* causes circular to irregular greyish lesions with dark margins (Damm et al., 2019) and *C. chlorophyti* causes intra‐ and interveinal necrotic lesions surrounded by slight chlorosis (Yang et al., 2012).

Except for *C. truncatum*, almost no information is available on the life cycle of other *Colletotrichum* spp. associated with soybean anthracnose. *C. truncatum* survives on seeds, crop residues, and weeds, and can form soybean‐infective microsclerotia (Hartman et al., 1986; Khan & Sinclair, 1991; Yang & Hartman, 2016). Although the role of weeds and alternative hosts in the epidemiology of the disease is still unclear, probably the main source of primary inoculum of *C. truncatum* and *C. plurivorum* is infected seeds, which contribute to dispersion over long distances and the introduction of new fungal isolates in an area (Dias et al., 2018; Hartman et al., 1999).

**FIGURE 4:**
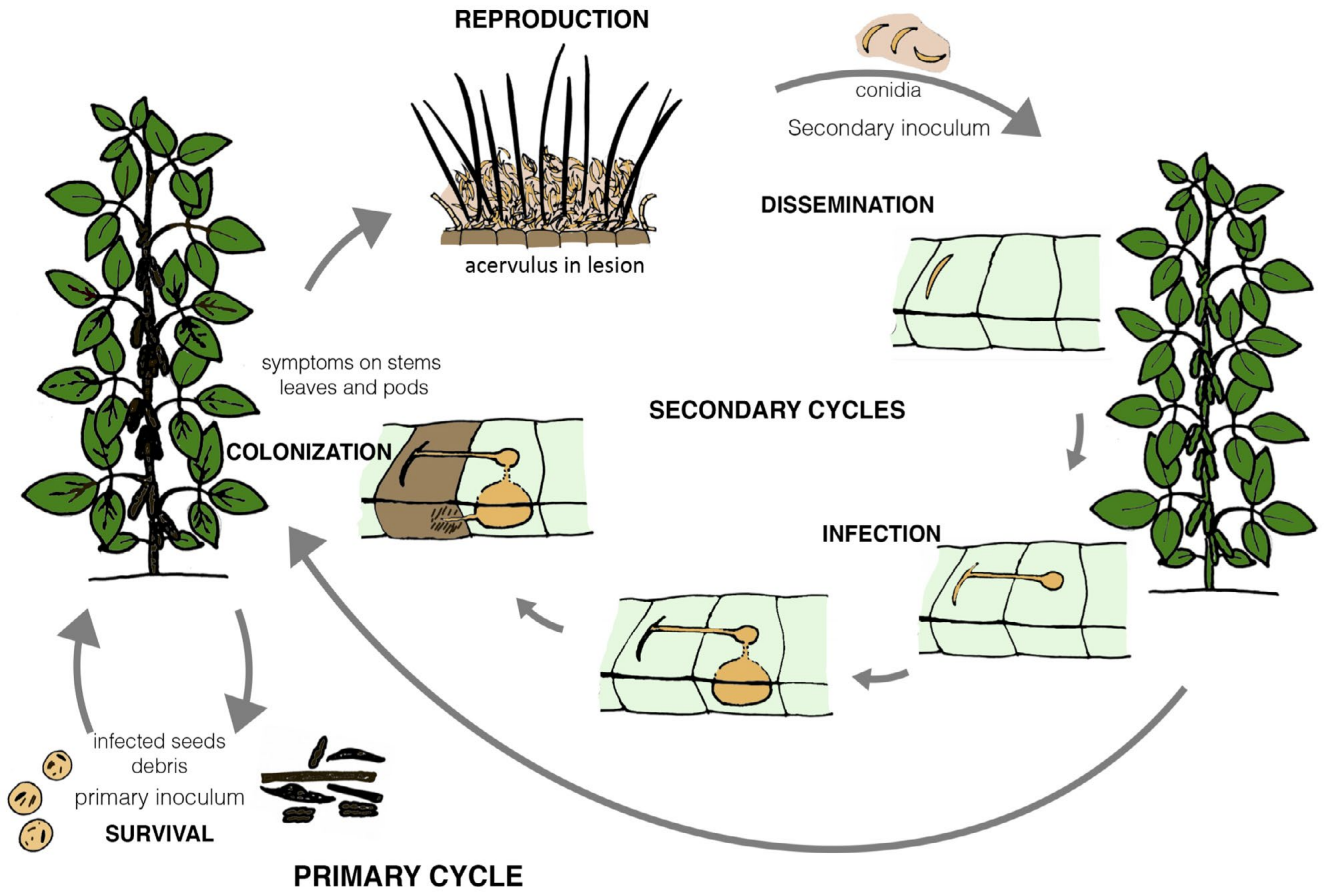
Disease cycle of soybean anthracnose caused by *Colletotrichum truncatum*

Fungal penetration occurs directly after conidial germination and formation of an appressorium on the plant surface. *C. truncatum* and *C. sojae* have the same patterns of infection and colonization of soybean leaves (Manandhar et al., 1985). *C. truncatum* has a hemibiotrophic lifestyle with a first stage during which the penetration peg develops into a typical primary hypha that is a biotrophic vesicle between the cell wall and the plasma membrane. The biotrophic phase is followed by a switch to the necrotrophic phase, with the production of secondary hyphae that colonize the tissue intra‐ and intercellularly, causing cell death (Bhadauria et al., 2013). The possibility of quiescent/endophytic infection is also reported (Bhadauria et al., 2013); consequently plant tissues can be infected without showing any symptoms (Chen et al., 2006). Symptoms of anthracnose appear during the necrotrophic stage, in which the formation of acervuli containing conidia occurs. The conidia represent the secondary inoculum of the disease, disseminated by water splash that dissolves the mucilage in which they are covered, and aids short‐range dispersal (Madden, 1997). The life cycle of *C. truncatum* is illustrated in Figure 4.

Understanding the relative importance of each s.c. in the development of the disease in the field is fundamental to direct epidemiological studies, which are essential for its effective management. Some species associated with soybean anthracnose, such as *C. sojae*, *C. plurivorum*, and *C. musicola*, have been reported to undergo the sexual state (Boufleur et al., 2020; Damm et al., 2019; Ramos et al., 2013). *Glomerella glycines* (now *C. sojae*) was considered the sexual morph of at least three species of *Colletotrichum* in the past, creating a taxonomic confusion that was solved by Damm et al. (2019), while others like *C. truncatum* and *C. destructivum* only occur in the asexual morph (Cannon et al., 2012; Damm et al., 2014). Can species with a sexual state have greater survivability on alternative hosts? Can genetic recombination play a role in survival or be an important source of variability in these species? Could sexual spores be spread differently from conidia? Do ascospores and conidia infect soybean tissues in the same way? These are some of the questions about the pathogen's epidemiology that still need answers as they directly impact the disease management.

## 4 DISEASE MANAGEMENT

The need for improvement of disease management motivated all efforts for a better understanding of soybean anthracnose proposed in this paper. The poor understanding of the life cycle and the epidemiological role of the *Colletotrichum* spp. associated with soybean may have led to ineffective disease management because it is not clear which species are responsible for the disease in the field.

Due to the potential for off‐season survival of species of *Colletotrichum* that infect soybean, and the long‐distance dissemination of the pathogen by seeds (Yang & Hartman, 2016), the management of soybean anthracnose should start with sowing disease‐free seeds (Pellegrino et al., 2010) and practising crop rotation. In most cases, seeds are symptomless; however, even low percentages of infection may lead to severe crop losses (Ciampi‐Guillardi et al., 2020; Pellegrino et al., 2010). To prevent the disease, seeds can be treated with systemic fungicides such as carboxanilide, dimethyldithiocarbamate, benzimidazoles, or triazoles (AGROFIT, 2020). Also, research on potential strategies of biological control with biopriming have been performed. Soybean seeds inoculated with *Pseudomonas aeruginosa* and *Trichoderma harzianum* reduced *C. truncatum* field incidence up to 92%, offering the same efficiency as the fungicide benomyl (Begum et al., 2010).

Currently, fungicides used as preventives are azoxystrobin, captan, mancozeb, carbendazim, thiophanate methyl, and members of the sterol demethylation inhibitors (DMI), such as triazoles (Dias et al., 2016; Nataraj et al., 2020; Poti et al., 2020). However, in recent years several studies have shown that fungicide efficiency is gradually reducing against soybean anthracnose (Dias et al., 2016; Poti et al., 2020).

In Brazil, when two seasons of soybean production were evaluated, chemical control with the use of triazoles combined with strobilurins was efficient during the first season, but not during the second season under natural *Colletotrichum* spp. infection (Dias et al., 2016). The resistance of *C. truncatum* isolates to multiple triazoles (flutriafol, fenbuconazole, tebuconazole, and metconazole) and reduced sensitivity to difenoconazole and propiconazole have been reported, indicating an inherent resistance as a result of *CYP51A* and *CYP51B* gene variations (Chen et al., 2018).

Carbendazim is a fungicide of the class of benzimidazole or methyl benzimidazole carbamate (MBC), which acts as a single‐site inhibitor (Oliver & Hewitt, 2014). Different studies showed that carbendazim is the most effective fungicide reducing *C. truncatum* growth in vitro (Agam et al., 2019; Ahamad et al., 2018; Kale & Barhate, 2016). During two seasons of soybean production in Nepal, in vivo trials showed that carbendazim (12%) combined with mancozeb (63%) reduced the disease incidence and increased the yield of the treated plots when compared with the control; this was considered the best treatment among the tested fungicides (Subedi et al., 2016). In contrast, in a study with 52 *C. truncatum* isolates from different fields, 89% of them were considered highly resistant to carbendazim (EC_50_ > 1,000 μg/ml) and 86% showed a mutation at codon 198 of the *TUB2* gene, which prevents a hydrogen bond between carbendazim and β‐tubulin and is highly correlated with resistant fungal strains (Cai et al., 2015; Poti et al., 2020).

Although studies on the efficiency of fungicides against anthracnose in the field look promising, it remains unclear which *Colletotrichum* species are responsible for the disease. This may explain the contradictory results found in different field studies. The loss of efficacy has made producers intensify fungicide applications and doses, increasing their costs and inducing stronger selective pressure on the pathogen (Poti et al., 2020). The correct identification of the causal agent of anthracnose is important to explain the real reason for the lack of efficiency of the active compounds in the field.

Besides the potential of losses due to infection by *Colletotrichum* species (Wrather et al., 2010), there have been no breeding programmes for soybean cultivars resistant to anthracnose until now (Yang & Hartman, 2015). The implementation of resistant cultivars can generally reduce production costs and so this is a more eco‐friendly solution when compared with chemical control (Talhinhas et al., 2016).

Anthracnose resistance genes tend to be highly specific, and the emergence of additional *Colletotrichum* species associated with the disease in soybean‐producing areas indicates the need to start focused programmes (Dias et al., 2018). The inheritance of resistance to soybean anthracnose caused by *C. truncatum* was demonstrated to be governed by more than one gene (Nataraj et al., 2020). In a test with 16 soybean accessions inoculated with a mixture of *C. truncatum* isolates, Dias et al. (2019) found that some soybean genetic materials with a high level of resistance in stems are highly susceptible to cotyledon infection. They hypothesized that the genetic resistance of cotyledons and stems might be under the control of genetically independent mechanisms. Sources of resistance of soybean to anthracnose caused by *C. truncatum* have been reported and studies in Brazil, India, and the USA revealed that 22 commercial cultivars, nine genotypes, and one soybean germplasm are highly resistant to *C. truncatum* (Costa et al., 2009; Dias et al., 2019; Nagaraj et al., 2014, 2020; Yang & Hartman, 2015). Research on sources of resistance to other *Colletotrichum* species associated with anthracnose has not yet been performed.

## 5 IDENTIFICATION AND MOLECULAR DIAGNOSTICS

Accurate identification of *Colletotrichum* strains to the species level is critical in plant pathology with regard to fungal detection in propagative host material, quarantine measures, selection of biocontrol agents, screening varieties in plant breeding, population genetics, and genomics (Jayawardena et al., 2016). If cryptic species are confused with a single species, the integrity and understanding of the species will be compromised (Batista et al., 2017), and the importance of this is fundamental in population genetics and genomic studies.

### 5.1 Morphological characters

Most *Colletotrichum* lineages pathogenic to soybean can be divided into two major groups based on morphology: those that have curved conidia, including *C. spaethianum*, *C. truncatum*, and *C. dematium* s.c., plus *C. chlorophyti* (Damm et al., 2009); and those with straight cylindrical conidia, including *C. gloeosporioides*, *C. gigasporum*, *C. magnum*, and *C. orchidearum* s.c. (Damm et al., 2012a, 2012b, 2019; Liu et al., 2014; Weir et al., 2012). The main character of the *C. acutatum* s.c. is cylindrical conidia with acute ends (Damm et al., 2012a), while the *C. boninense* s.c. have straight cylindrical to clavate conidia (Damm et al., 2012b). Except for the *C. gigasporum* s.c., which have a distinctive morphological feature, with conidia up to 32 μm long and average length 26 μm (Liu et al., 2014), morphological characters overlap between species and s.c. associated with soybean, and have been fully described before (Damm et al., 2009, 2012a, 2012b, 2019; Liu et al., 2014; Weir et al., 2012); therefore, they should not be used for identification of *Colletotrichum*.

### 5.2 Molecular identification

Considering the importance of the *Colletotrichum* genus as a plant pathogen worldwide, rapid identification of a large collection of *Colletotrichum* isolates is often required. However, there is no consensus on the best molecular markers to discriminate species in each *Colletotrichum* s.c. (Vieira et al., 2020). Currently, there is no minimum or optimal standard set of molecular markers able to discriminate all the *Colletotrichum* s.c. (Marin‐Felix et al., 2017; Vieira et al., 2020). In general, five markers are amongst those commonly used to differentiate species among the distinct *Colletotrichum* s.c., especially those associated with soybean anthracnose: ITS, *GAPDH*, *TUB2*, *CHS‐1*, and *ACT* (Damm et al., 2009, 2019). Species within the *C. acutatum* s.c. can be effectively differentiated by both *TUB2* and *GAPDH* markers (Damm et al., 2012a), while *GAPDH* alone can recognize all species within the *C*. *boninense* s.c. (Damm et al., 2012b). Combined gene analysis of ITS, *GAPDH*, *CHS‐1*, *ACT*, and *TUB2* sequences can identify all the species within both the *C*. *dematium* and *C. gigasporum* s.c. (Liu et al., 2014). For the *C. truncatum* s.c. *GAPDH* is the most informative marker, followed by *TUB2* and *ACT* (Vieira et al., 2020); for *C. spaethianum* and *C. truncatum* s.c., the inclusion of *HIS3* in the multilocus combination is needed for the precise discrimination of species, whereas this locus is not informative for other complexes (Jayawardena et al., 2016). The combination of ITS, *GAPDH*, *CHS‐1*, *HIS3*, *ACT*, and *TUB2* can differentiate species within the *C. orchidearum* s.c. (Damm et al., 2019). Species within the *C. gloeosporioides* s.c. can be distinguished by a combination of *ApMat* and *GS* sequences (Liu et al., 2015).

Because it might be unrealistic for most researchers to sequence multiple loci across a large set of isolates, it is useful to recommend markers with more phylogenetic informativeness (Vieira et al., 2020). In the majority of the 14 *Colletotrichum* species complexes *GAPDH*, *HIS3*, and *TUB2* were found to be the most variable and informative markers for discriminating species.

The consensus is that species identification should not be based on BLAST searches of individual fungal sequences on NCBI/GenBank, but instead on robust phylogenetic analyses based on the concordance of multiple gene genealogies, including sequences from type species (Cannon et al., 2012). One reason is that most molecular markers alone do not exhibit sufficient polymorphism to discriminate *Colletotrichum* species, mainly within complexes, so that variation level among sequences is low. This is especially problematic in species with similar morphological characters that can be easily confused. Another issue is the problem of misidentification in the sequences deposited in NCBI as mentioned before, most likely as a consequence of the recent taxonomic reassessment of the genus that led to a massive increase in incorrectly assigned ITS sequences (Rogério et al., 2017). The ITS region should not be used singly to describe new *Colletotrichum* taxa because there is not enough discrimination for resolving the taxonomy.

Beyond phylogenetic trees, haplotype networks of concatenated sequences could be employed to infer geographical patterns of distribution or even host association among fungal lineages, below the species level. This approach was efficiently carried out in the identification of distinct clusters in *C. truncatum* strains causing soybean anthracnose in Brazil, by identifying groups of lineages associated with other Fabaceae hosts and weeds as well (Rogério et al., 2017).

### 5.3 Molecular diagnosis

In general, molecular diagnostic tests are developed on the basis of molecular markers largely used in phylogenetic studies. Despite some caveats, the most widely used molecular marker to design specific primers to detect fungal pathogens is the nuclear ribosomal cluster (Mancini et al., 2016; Pecchia et al., 2019). Recent studies have pointed out the lack of variation in the ITS region needed to develop specific primers for most *Colletotrichum* species (Da Lio et al., 2018). However, the intergenic spacer (IGS) region can be an alternative to ITS because it tends to contain more polymorphic sites and has proved to be an efficient marker for detecting *C. lupini* in lupins by PCR and could, therefore, be considered as an alternative target for other *Colletotrichum* species (Pecchia et al., 2019).

Molecular diagnostic techniques based on the detection of fungal DNA have been widely used for species‐specific detection of *Colletotrichum* associated with soybean anthracnose. PCR is the method of choice in the field of molecular diagnosis of soybean pathogens, as it enables an exponential amplification of the target DNA sequence, making it a fast, efficient, and attractive technique (Kumar et al., 2020). Using these molecular techniques, tiny amounts of host samples are sufficient for the detection of *Colletotrichum* in soybean seeds or other plant tissues. Several PCR‐based strategies are available for these purposes, such as multiplex PCR, loop‐mediated isothermal amplification (LAMP), real‐time or quantitative PCR (qPCR), and droplet digital PCR (ddPCR), among others, using specific primer pairs and sometimes excluding the need for DNA extraction (Ciampi‐Guillardi et al., 2020; Tian et al., 2017; Wang et al., 2017).

Multiplex qPCR assays have been consistently used for pathogen diagnosis in plant material by allowing the simultaneous amplification of multiple DNA targets in a single reaction (Schena et al., 2017). A highly sensitive multiplex TaqMan qPCR assay targeting the *GAPDH* gene was developed to detect and quantify as little as 0.3 pg of *C. truncatum* DNA, along with two other pathogens in soybean seeds (Ciampi‐Guillardi et al., 2020). The method was able to access fungal DNA directly from seed soaking solution, amplifying only the target species and not any other fungi commonly associated with soybean seeds. The high specificity of the assay is provided by the internal TaqMan probes, which overcomes the risk of false positives and/or false negatives. For the diagnosis of *C. truncatum*, a multiplex qPCR assay targeting the *cox1* gene has also been proposed to distinguish four *Colletotrichum* species infecting soybean, *C. chlorophyti*, *C. sojae*, *C. incanum*, and *C. truncatum*, by using two duplex sets based on melting point temperatures. While successful detection was achieved with 0.1 pg of *C. truncatum* DNA, the assay may not be suitable for field diagnostics because it was tested only on purified *Colletotrichum* DNA, not on host tissue samples (Yang et al., 2015). The correct identification of *Colletotrichum* spp. in seeds is essential for diagnostic laboratories and producers, avoiding the introduction and dissemination of the pathogen in soybean fields (Ramiro et al., 2019). To date, there are still no diagnostic tests for all *Colletotrichum* species associated with soybean.

New tools have been developed to quickly detect *Colletotrichum* DNA in host samples. LAMP is a new nucleic acid amplification technology that enables the synthesis of large amounts of DNA in a short period of time with high specificity **(**Fu et al., 2011; Notomi et al., 2000). It could be a potential alternative to PCR because the LAMP protocol does not require a thermocycler. Despite the great potential attributed to the technique, LAMP has not been widely used for detecting *Colletotrichum* species associated with soybean anthracnose so far. Rapid LAMP diagnostic assays were proposed to detect *C. truncatum*, targeting the large subunit of RNA polymerase II (*Rpb1*) coding gene (Tian et al., 2017), and *C. gloeosporioides*, whose target was a glutamine synthetase (*GS*) gene (Wang et al., 2017) in soybean samples. For *C. truncatum* the detection limit of the LAMP assay was 100 pg/μl of fungal DNA per reaction, a hundred times greater than the amount detected in the qPCR assay proposed by Tian et al. (2017) and more than a thousand times less sensitive than the qPCR assay developed by Ciampi‐Guillardi et al. (2020).

An alternative approach would be to identify genomic regions specific to emerging *Colletotrichum* species or even to particular lineages using a computational approach based on whole‐genome comparison of distinct isolates or lineages. This approach has been successfully used to develop specific markers for the detection of *C. lupini* and other plant pathogens (Pecchia et al., 2019; Pieck et al., 2017; Thierry et al., 2020). Specific care must be taken in cases of recently diverged taxa, bearing in mind that it is unlikely that a single genomic region would perfectly meet all the requirements of a specific detection, especially in fungal lineages with very low divergence levels and recent genetic exchanges between them (Thierry et al., 2020).

## 6 POPULATION GENETIC STUDIES

Genetic variability in the form of the presence of different alleles occurring at different frequencies in genes is crucial to provide greater endurance to environmental changes and to increase species local adaptation over time (Barrett & Schluter, 2008; Hartl & Clark, 1997). Genetic investigation using a group of individuals provides a broader overview of species variability than a study with few individuals (McDonald, 1997) and can be used to make inferences about the predominant mode of pathogen reproduction and their impact on genotypic diversity (McDonald & Linde, 2002). In recent years, plant pathologists have been interested in investigations of genetic variation in pathogen populations, providing tremendous insights into the biology of fungal plant parasites (Giraud et al., 2008).

Knowledge of genetic structure, that is, the amount and distribution of genetic variation within and among populations, allows us to investigate the evolutionary forces (gene flow, genetic drift, mutation, and natural selection) acting as modulators of genetic diversity in populations (Giraud et al., 2008). The evolutionary potential of pathogen populations is directly guided by their genetic diversity (Croll & Laine, 2016). Thus, knowledge of the genetic structure gives information about the evolutionary processes that influenced plant pathogen populations in the past and provides insights into their future evolutionary potential (McDonald & Linde, 2002). Such information could be useful to optimize the management of resistance genes and fungicides in agriculture, and therefore to control plant diseases more effectively (Zhan, 2009).

Several population genetic studies of *Colletotrichum* species have been published over the years, and these investigations have increased our knowledge of the genetic variation of many important agricultural species (Banniza et al., 2018; Baroncelli et al., 2015; Ciampi‐Guillardi et al., 2014; Crouch, Tredway, et al., 2009; Ureña‐Padilla et al., 2002; Xavier et al., 2018). Despite the importance of soybean anthracnose, few studies are available on this pathosystem, and population genetic studies are even more scarce. Previous research mainly focused on genetic differences among *C. truncatum* isolates obtained from a range of hosts using distinct types of genetic markers, which revealed high genetic diversity and possible genetic recombination (Ford et al., 2004; Katoch et al., 2017; Ranathunge et al., 2009; Rogério et al., 2017; Sant’anna et al., 2010; Sharma, 2009; Vasconcelos et al., 1994;).

As *C. truncatum* was the only fungal species associated with soybean anthracnose in Brazil up to 2007 (Rogério et al., 2017), an investigation of the genetic structure of *C. truncatum* populations in the main soybean production areas was performed (Rogério et al., 2019). High levels of genetic diversity within populations and no evidence of intraregional gene flow were revealed by microsatellite data. This study also suggested that Brazilian *C. truncatum* populations resulted from at least three founder events, which led to three genetic groups that spread throughout the country, conserving syntopy (Rogério et al., 2019). Another study investigated populations of *C. truncatum* from Brazil and Argentina and identified that intragroup similarity was greater among the Argentinian isolates than the Brazilian group, with a strong correlation between geographical origin and genetic grouping (Dias et al., 2019). Such a large difference in genetic diversity was associated with a greater geographic breadth of the sampling in Brazilian populations, in addition to a greater genetic variability of host cultivars, which could be reflected in the variability of the isolates among Brazilian regions.

Considering the increase of soybean anthracnose in South America, population studies suggest an association between the inherent variability of the pathogen and the climatic and cultural features, as well the genetic makeup of commercial soybean cultivars used (Dias et al., 2019; Rogério et al., 2019). These population studies highlight intraspecific pathogen variability as a major feature in genetic breeding for anthracnose resistance. Efforts in breeding programmes aiming at anthracnose resistance should take into account the population structure and the genetic diversity levels of the pathogen by using representative isolates of the genetic variability of the species for screening soybean resistant cultivars.

Although there have been advances in understanding the genetic variation in *C. truncatum* infecting soybean and its impact on disease management strategies, many gaps have not yet been filled. Expanding the discrimination of genetic groups recently detected and the estimation of recombination rates may provide a potent approach to elucidate the pathogen life history and to address fundamental questions about the evolution and demographic history of this species (Rogério et al., 2019; Stukenbrock, 2016).

## 7 GENOMICS AS A TOOL FOR UNDERSTANDING PATHOGENICITY FACTORS

Recent technological advances in next‐generation sequencing and computational tools have made it possible to sequence and analyse whole genomes of many plant pathogens (Sant’Anna et al., 2010). These technologies continue to advance rapidly, and costs have declined to the point that it is becoming affordable to sequence genomes of many individuals within a species (Grünwald et al., 2016; Raffaele & Kamoun, 2012). This genomic revolution provides a major opportunity to connect the gaps between molecular biology, evolutionary genetics, and epidemiology (Plissonneau et al., 2017), playing a key role in plant disease management strategies (Klosterman et al., 2016).

The availability of a large number of genetic markers distributed throughout the genome enables the refinement of molecular variation investigations. The use of these markers provides fine‐grained resolution of genetic divergence, recombination, demography, as well as evolutionary biology of pathogen populations, enabling more robust inferences compared to studies based on a limited number of genetic markers (Brumfield et al., 2003; Helyar et al., 2011; Luikart et al., 2003). Population genomics analyses of a large number of loci offer an excellent opportunity to determine the genetic basis of many fungal phenotypes, including virulence (Plissonneau et al., 2017; Sarrocco et al., 2020). Furthermore, techniques such as genome‐wide association studies (GWAS), quantitative trait locus (QTL) mapping, and genome scans for signatures of selection and selective sweeps are powerful tools to identify genes involved in host‐specific interactions of fungal pathogens (Grünwald et al., 2016; Plissonneau et al., 2017).

To date, four *Colletotrichum* genomes isolated from soybean plants with symptoms have been sequenced: *C. truncatum* (IMI 507125), *C. plurivorum* (IMI 507127), *C. musicola* (IMI 507128), and *C. sojae* (IMI 507126) (Rogério et al., 2020). Additionally, the genome of two other strains of *C. truncatum*, MTCC 3114 and TYU, isolated from *Capsicum annuum* and *Taxus cuspidata,* respectively, and a strain of *C. chlorophyti* (NTL11) from tomato (*Solanum lycopersicon*) are currently available (Gan et al., 2017; Rao & Nandineni, 2017; Rogério et al., 2020). A summary of the genome assembly statistics is available in Table 2. The pathogenicity of the four *Colletotrichum* spp. sequenced by Rogério et al. (2020) to soybean fulfilled Koch's postulates on soybean. While the other sequenced isolates (MTCC 3114, TYU, and NTL11) belong to species pathogenic to soybean, it was not confirmed if they can infect soybean. The genome sequence data of *Colletotrichum* species pathogenic to soybean currently available may greatly aid our understanding of host–pathogen interactions besides offering a useful resource for further research in comparative genomics and evolutionary studies of *Colletotrichum*.

**TABLE 2:** Summary of *Colletotrichum* spp. pathogenic to soybean that have whole genome sequences

Species	Strain	Host	Country	Accession no.	Assembly length (Mb)	GC (%)	No. of predicted genes	Reference
*C. chlorophyti*	NTL11	*Solanum lycopersicon*	Japan	MPGH00000000	52.40	50.06	10,419	Gan et al., 2017
*C. musicola*	IMI 507128	*Glycine max*	Brazil	WIGM00000000	52.73	54.97	16,826	Rogério et al., 2020
*C. plurivorum*	IMI 507127	*G. max*	Brazil	WIGO00000000	49.70	55.86	16,153	Rogério et al., 2020
*C. sojae*	IMI 507126	*G. max*	Brazil	WIGN00000000	49.35	55.92	16,124	Rogério et al., 2020
*C. truncatum*	IMI 507125	*G. max*	Brazil	VUJX00000000	56.10	50.12	15,901	Rogério et al., 2020
*C. truncatum*	MTCC 3114	*Capsicum annum*	India	NBAU00000000	55.30	49.61	13,724	Rao & Nandineni, 2017
*C. truncatum*	TYU	*Taxus cuspidata*	South Korea	NOWE00000000	53.00	49.61	–	–
*C. truncatum*	GO2−03	*G. max*	Brazil	SRX7095355	–	–	–	Rogério et al., 2019
*C. truncatum*	MT5−32	*G. max*	Brazil	SRX7095354	–	–	–	Rogério et al., 2019
*C. truncatum*	MT5−26	*G. max*	Brazil	SRX7095353	–	–	–	Rogério et al., 2019
*C. truncatum*	MT5−12	*G. max*	Brazil	SRX7095352	–	–	–	Rogério et al., 2019
*C. truncatum*	MT4−13	*G. max*	Brazil	SRX7095351	–	–	–	Rogério et al., 2019
*C. truncatum*	MT4−05	*G. max*	Brazil	SRX7095350	–	–	–	Rogério et al., 2019
*C. truncatum*	MT3−21	*G. max*	Brazil	SRX7095349	–	–	–	Rogério et al., 2019
*C. truncatum*	MT3−01	*G. max*	Brazil	SRX7095348	–	–	–	Rogério et al., 2019
*C. truncatum*	GO5−25	*G. max*	Brazil	SRX7095347	–	–	–	Rogério et al., 2019
*C. truncatum*	GO5−14	*G. max*	Brazil	SRX7095346	–	–	–	Rogério et al., 2019
*C. truncatum*	GO5−11	*G. max*	Brazil	SRX7095345	–	–	–	Rogério et al., 2019
*C. truncatum*	GO4−17	*G. max*	Brazil	SRX7095344	–	–	–	Rogério et al., 2019
*C. truncatum*	GO4−08	*G. max*	Brazil	SRX7095343	–	–	–	Rogério et al., 2019
*C. truncatum*	GO4−07	*G. max*	Brazil	SRX7095342	–	–	–	Rogério et al., 2019
*C. truncatum*	GO2−12	*G. max*	Brazil	SRX7095341	–	–	–	Rogério et al., 2019
*C. truncatum*	GO2−06	*G. max*	Brazil	SRX7095340	–	–	–	Rogério et al., 2019
*C. truncatum*	MT2−05	*G. max*	Brazil	SRX7095339	–	–	–	Rogério et al., 2019
*C. truncatum*	MT1−01	*G. max*	Brazil	SRX7095338	–	–	–	Rogério et al., 2019

Scanning the *Colletotrichum* genomes for identification of full putative effector repertoires of the pathogen may be a useful tool for soybean breeders in the development of new cultivars with durable resistance against anthracnose (Barsoum et al., 2019; Lenman et al., 2016; Oliver & Solomon, 2010; Prasad et al., 2019; Van de Wouw & Idnurm, 2019). Although there is no breeding programme aimed at screening resistance to soybean anthracnose so far, the genomic resources now available may support the development of future programmes. Additionally, genome resources can be used to accelerate the development of diagnostic tools for plant pathogens (Klosterman et al., 2016), impacting the application of more accurate management strategies.

Advances in comparative genomics and population genomic approaches open new perspectives to increase our understanding of the molecular mechanisms underpinning the pathogenesis and adaptive processes of these pathogens (Klosterman et al., 2016; Stukenbrock & Bataillon, 2012). Mechanisms regarding *C. truncatum* pathogenicity have been investigated to understand the factors involved in the pathogenesis of different hosts (Auyong, 2015; Auyong et al., 2012; Madden, 1997; Ranathunge et al., 2009). With the availability of genomic sequences for *Colletotrichum* species pathogenic to soybean, new advances into pathogenic processes at the molecular level are possible, contributing to improving our knowledge in the host–fungal interactions in the soybean–anthracnose pathosystem, and thus developing effective and novel strategies to combat the pathogens.

## 8 CONCLUSIONS AND FUTURE PERSPECTIVES

Knowing precisely the diversity of a pathogen is crucial from taxonomic, biological, and ecological standpoints. Indeed, pathogen identity has direct implications for disease management by cultural or chemical strategies as well as for disease resistance breeding programmes. Furthermore, an effective management of new *Colletotrichum* species requires tools to discriminate between emerging and established fungal populations associated with soybean, aiming to detect the pathogens at the earliest point to monitor and limit their spread.

*C. truncatum* has been considered the most important causal agent of soybean anthracnose. However, our survey showed that at least 12 *Colletotrichum* lineages are associated with soybean, with the *C*. *truncatum* and *C. orchidearum* s.c. having the greatest impact and the broadest worldwide distribution. Most of the information available on soybean anthracnose until now has been limited to *C. truncatum*. Taking into account the numerous *Colletotrichum* species causing the disease, there is a gap in the knowledge of epidemiology, worldwide movement, distribution, identification, control measures, fungicide efficiency, and genetic resistance for all of the species.

In agreement with Vieira et al. (2020), more robust genomic sampling is required to improve our understanding of relationships among taxa in the genus *Colletotrichum*, and also our ability to distinguish species within s.c. Genome data is now available for several *Colletotrichum* species associated with anthracnose in soybean, such as *C. truncatum*, *C. musicola*, *C. plurivorum*, *C. sojae*, and *C. chlorophyti* (Gan et al., 2017; Rao & Nandineni, 2017; Rogério et al., 2020), yet a comprehensive phylogenomic study of the genus is still needed. A population genomics approach and comparative genomics investigations can be used to identify candidate genes involved in pathogenicity, virulence (or aggressiveness), host specialization, fungicide resistance, and adaptation to different environments with higher precision, contributing to a better understanding of *Colletotrichum* species dynamic in the agroecosystems.

## CONFLICTS OF INTEREST

The authors declare no conflict of interest.

## Supporting information


**TABLE S1** Strains of *Colletotrichum* spp. studied, with collection details and GenBank accession numbers

## Data Availability

Data sharing is not applicable as no new data were generated or analysed.

## References

[mpp13036-bib-0001] Agam, M.N. , Raut, R.A. , Jejurkar, G.B. & Sable, S.B. (2019) Evaluation of the fungicides, botanicals and bioagents against *Colletotrichum truncatum* causing anthracnose of soybean in pot culture. Journal of Pharmacognosy and Phytochemistry, 8, 629–634.

[mpp13036-bib-0002] AGROFIT (2020). Sistemas de agrotóxicos fitossanitários. Available at: http://extranet.agricultura.gov.br/agrofit_cons/principal_agrofit_cons [accessed August 2020].

[mpp13036-bib-0003] Ahamad, V. , Kaintura, P. & Gaur, G. (2018) In vitro evaluation of fungicides and bio‐control agents against *Colletotrichum truncatum* in soybean in Dehradun Valley. International Journal of Pure and Applied Bioscience, 6, 869–874.

[mpp13036-bib-0004] Auyong, A.S. (2015) The role of cutinase and its impact on pathogenicity of *Colletotrichum truncatum* . Journal of Plant Pathology and Microbiology, 6, 1000259.

[mpp13036-bib-0005] Auyong, A.S.M. , Ford, R. & Taylor, P.W.J. (2012) Genetic transformation of *Colletotrichum truncatum* associated with anthracnose disease of chili by random insertional mutagenesis. Journal of Basic Microbiology, 52, 372–382. 22052577 10.1002/jobm.201100250

[mpp13036-bib-0006] Banniza, S. , Warale, R. , Menat, J. , Cohen‐Skali, A. , Armstrong‐Cho, C. & Bhadauria, V. (2018) The long path to understanding the host–pathogen interactions of *Colletotrichum lentis* on lentil. Canadian Journal of Plant Pathology, 40, 199–209.

[mpp13036-bib-0007] Barbieri, M.C.G. , Ciampi‐Guillardi, M. , Moraes, S.R.G. , Bonaldo, S.M. , Rogério, F. , Linhares, R.R. et al. (2017) First report of *Colletotrichum cliviae* causing anthracnose on soybean in Brazil. Plant Disease, 101, 1677.

[mpp13036-bib-0008] Baroncelli, R. , Talhinhas, P. , Pensec, F. , Sukno, S.A. , Le Floch, G. & Thon, M.R. (2017) The *Colletotrichum acutatum* species complex as a model system to study evolution and host specialization in plant pathogens. Frontiers in Microbiology, 8, 2001. 29075253 10.3389/fmicb.2017.02001 PMC5641571

[mpp13036-bib-0009] Baroncelli, R. , Zapparata, A. , Sarrocco, S. , Sukno, S.A. , Lane, C.R. , Thon, M.R. et al. (2015) Molecular diversity of anthracnose pathogen populations associated with UK strawberry production suggests multiple introductions of three different *Colletotrichum species* . PLoS One, 10, e0129140. 26086351 10.1371/journal.pone.0129140 PMC4472692

[mpp13036-bib-0010] Barrett, R. & Schluter, D. (2008) Adaptation from standing genetic variation. Trends in Ecology and Evolution, 23, 38–44. 18006185 10.1016/j.tree.2007.09.008

[mpp13036-bib-0011] Barsoum, M. , Sabelleck, B. , Spanu, D.P. & Panstruga, R. (2019) Rumble in the effector jungle: Candidate effector proteins in interactions of plants with powdery mildew and rust fungi. Critical Reviews in Plant Sciences, 38, 255–279.

[mpp13036-bib-0012] Batista, D. , Silva, D.N. , Vieira, A. , Cabral, A. , Pires, A.S. , Loureiro, A. et al. (2017) Legitimacy and implications of reducing *Colletotrichum kahawae* to subspecies in plant pathology. Frontiers in Plant Science, 7, 2051. 28119726 10.3389/fpls.2016.02051 PMC5220086

[mpp13036-bib-0137] Batzer, J.C. & Mueller, D.S. (2020) Soybean fungal endophytes *Alternaria* and *Diaporthe* spp. are differentially impacted by fungicide application. Plant Disease, 104, 52–59. 31738691 10.1094/PDIS-05-19-1001-RE

[mpp13036-bib-0013] Begum, M.M. , Sariah, M. , Puteh, A.B. , Zainal Abidin, M.A. , Rahman, M.A. & Siddiqui, Y. (2010) Field performance of bio‐primed seeds to suppress *Colletotrichum truncatum* causing damping‐off and seedling stand of soybean. Biological Control, 53, 18–23.

[mpp13036-bib-0014] Bhadauria, V. , Banniza, S. , Vandenberg, A. , Selvaraj, G. & Wei, Y. (2013) Overexpression of a novel biotrophy‐specific *Colletotrichum truncatum* effector, CtNUDIX, in hemibiotrophic fungal phytopathogens causes incompatibility with their host plants. Eukaryotic Cell, 12, 2–11. 22962277 10.1128/EC.00192-12 PMC3535838

[mpp13036-bib-0015] Boufleur, T.R. , Castro, R.R.L. , Rogério, F. , Ciampi‐Guillardi, M. , Baroncelli, R. & Massola Júnior, N.S. (2020) First report of *Colletotrichum musicola* causing soybean anthracnose in Brazil. Plant Disease, 104, 1858.

[mpp13036-bib-0016] Brumfield, R.T. , Beerli, P. , Nickerson, D.A. & Edwards, S.V. (2003) The utility of single nucleotide polymorphisms in inferences of population history. Trends in Ecology and Evolution, 18, 249–256.

[mpp13036-bib-0017] Cai, L. , Hyde, K.D. , Taylor, P.W.J. , Weir, B.S. , Waller, J.M. , Abang, M.M. et al. (2009) A polyphasic approach for studying *Colletotrichum* . Fungal Diversity, 39, 183–204.

[mpp13036-bib-0018] Cai, M. , Lin, D. , Chen, L. , Bi, Y. , Xiao, L. & Liu, X. (2015) M233I mutation in the β‐tubulin of *Botrytis cinerea* confers resistance to zoxamide. Scientific Reports, 5, 16881. 26596626 10.1038/srep16881 PMC4657022

[mpp13036-bib-0019] Cannon, P.F. , Damm, U. , Johnston, P.R. & Weir, B.S. (2012) *Colletotrichum* – current status and future directions. Studies in Mycology, 73, 181–213. 23136460 10.3114/sim0014 PMC3458418

[mpp13036-bib-0020] Chen, L.S. , Chu, C. , Liu, C.D. , Chen, R.S. & Tsay, J.G. (2006) PCR‐based detection and differentiation of anthracnose pathogens, *Colletotrichum gloeosporioides* and *C. truncatum*, from vegetable soybean in Taiwan: PCR‐based detection of *Glomerella/Colletotrichum* spp. Journal of Phytopathology, 154, 654–662.

[mpp13036-bib-0021] Chen, S. , Wang, Y. , Schnabel, G. , Peng, C.A. , Lagishetty, S. , Smith, K. et al. (2018) Inherent resistance to 14α‐demethylation inhibitor fungicides in *Colletotrichum truncatum* is likely linked to *CYP51A* and/or *CYP51B* gene variants. Phytopathology, 108, 1263–1275. 29792573 10.1094/PHYTO-02-18-0054-R

[mpp13036-bib-0022] Ciampi‐Guillardi, M. , Baldauf, C. , Souza, A.P. , Silva‐Junior, G.J. & Amorim, L. (2014) Recent introduction and recombination in *Colletotrichum acutatum* populations associated with citrus postbloom fruit drop epidemics in São Paulo, Brazil. Phytopathology, 104, 769–778. 24423403 10.1094/PHYTO-06-13-0165-R

[mpp13036-bib-0023] Ciampi‐Guillardi, M. , Ramiro, J. , de Moraes, M.H.D. , Barbieri, M.C.G. & Massola, N.S. (2020) Multiplex qPCR assay for direct detection and quantification of *Colletotrichum truncatum*, *Corynespora cassiicola*, and *Sclerotinia sclerotiorum* in soybean seeds. Plant Disease, 104, 3002–3009. 32822262 10.1094/PDIS-02-20-0231-RE

[mpp13036-bib-0024] Costa, I.F.D. , Balardin, R.S. , Medeiros, L.A.M. , Lenz, G. , Gulart, C.A. , Zemolin, C.R. et al. (2009) Reação de germoplasma comercial de soja a *Colletotrichum truncatum* . Tropical Plant Pathology, 34, 47–50.

[mpp13036-bib-0025] Croll, D. & Laine, A.‐L. (2016) What the population genetic structures of host and pathogen tell us about disease evolution. New Phytologist, 212, 537–539. 10.1111/nph.14203 27735071

[mpp13036-bib-0026] Crouch, J.A. , Clarke, B.B. & Hillman, B.I. (2009) What is the value of ITS sequence data in *Colletotrichum* systematics and species diagnosis? A case study using the falcate‐spored graminicolous *Colletotrichum* group. Mycologia, 101, 648–656. 19750944 10.3852/08-231

[mpp13036-bib-0027] Crouch, J.A. , Tredway, L.P. , Clarke, B.B. & Hillman, B.I. (2009) Phylogenetic and population genetic divergence correspond with habitat for the pathogen *Colletotrichum cereale* and allied taxa across diverse grass communities. Molecular Ecology, 18, 123–135. 19076279 10.1111/j.1365-294X.2008.04008.x

[mpp13036-bib-0028] Da Lio, D. , Cobo‐Díaz, J.F. , Masson, C. , Chalopin, M. , Kebe, D. , Giraud, M. et al. (2018) Combined metabarcoding and multi‐locus approach for genetic characterization of *Colletotrichum* species associated with common walnut (*Juglans regia*) anthracnose in France. Scientific Reports, 8, 10765. 30018385 10.1038/s41598-018-29027-z PMC6050315

[mpp13036-bib-0030] Damm, U. , Cannon, P.F. , Woudenberg, J.H.C. & Crous, P.W. (2012a) The *Colletotrichum acutatum* species complex. Studies in Mycology, 73, 37–113. 23136458 10.3114/sim0010 PMC3458416

[mpp13036-bib-0031] Damm, U. , Cannon, P.F. , Woudenberg, J. , Johnston, P.R. , Weir, B.S. , Tan, Y.P. et al. (2012b) The *Colletotrichum boninense* species complex. Studies in Mycology, 73, 1–36. 23136457 10.3114/sim0002 PMC3458415

[mpp13036-bib-0032] Damm, U. , O’Connell, R.J. , Groenewald, J.Z. & Crous, P.W. (2014) The *Colletotrichum destructivum* species complex – hemibiotrophic pathogens of forage and field crops. Studies in Mycology, 79, 49–84. 25492986 10.1016/j.simyco.2014.09.003 PMC4255528

[mpp13036-bib-0033] Damm, U. , Sato, T. , Alizadeh, A. , Groenewald, J.Z. & Crous, P.W. (2019) The *Colletotrichum dracaenophilum*, *C. magnum* and *C. orchidearum* species complexes. Studies in Mycology, 92, 1–46. 29997400 10.1016/j.simyco.2018.04.001 PMC6030544

[mpp13036-bib-0034] Damm, U. , Wounderberg, J.H.C. , Cannon, P.F. & Crous, P.W. (2009) *Colletotrichum* species with curved conidia from herbaceous hosts. Fungal Diversity, 39, 45–87.

[mpp13036-bib-0036] Dean, R. , van Kan, J.A.L. , Pretorius, Z.A. , Hammond‐Kosack, K.E. , di Pietro, A. , Spanu, P.D. et al. (2012) The Top 10 fungal pathogens in molecular plant pathology: Top 10 fungal pathogens. Molecular Plant Pathology, 13, 414–430. 22471698 10.1111/j.1364-3703.2011.00783.x PMC6638784

[mpp13036-bib-0037] Dias, M.D. , Dias‐Neto, J.J. , Santos, M.D.M. , Formento, A.N. , Bizerra, L.V.A.S. , Fonseca, M.E.N. et al. (2019) Current status of soybean anthracnose associated with *Colletotrichum truncatum* in Brazil and Argentina. Plants, 8, 459. 10.3390/plants8110459 PMC6918314 31671821

[mpp13036-bib-0038] Dias, M.D. , Fonseca, M.E.N. , Dias‐Neto, J.J. , Santos, M.D.M. , Pandolfo, G.M. , Boiteux, L.S. et al. (2018) Biology, pathogenicity, and haplotype analyses of *Colletotrichum cliviae*: A novel soybean anthracnose agent in warm tropical areas. Tropical Plant Pathology, 43, 439–451.

[mpp13036-bib-0039] Dias, M.D. , Pinheiro, V.F. & Café‐Filho, A.C. (2016) Impact of anthracnose on the yield of soybean subjected to chemical control in the north region of Brazil. Summa Phytopathologica, 42, 18–23.

[mpp13036-bib-0040] FAOSTAT (Food and Agriculture Organization of the United Nations) (2018) Food and agriculture data. Available at: http://www.fao.org/faostat/en [accessed August 2020].

[mpp13036-bib-0041] Ford, R. , Banniza, S. , Phaotita, W. & Taylor, P.W.J. (2004) Morphological and molecular discrimination of *Colletotrichum truncatum* causing anthracnose on lentil in Canada. Australasian Plant Pathology, 33, 559–569.

[mpp13036-bib-0042] Fu, S. , Qu, G. , Guo, S. , Ma, L. , Zhang, N.A. , Zhang, S. et al. (2011) Applications of loop‐mediated isothermal DNA amplification. Applied Biochemistry and Biotechnology, 163, 845–850. 20844984 10.1007/s12010-010-9088-8

[mpp13036-bib-0043] Gan, P. , Narusaka, M. , Tsushima, A. , Narusaka, Y. , Takano, Y. & Shirasu, K. (2017) Draft genome assembly of *Colletotrichum chlorophyti*, a pathogen of herbaceous plants. Genome Announcements, 5, e01733–16. 28280027 10.1128/genomeA.01733-16 PMC5347247

[mpp13036-bib-0044] Giraud, T. , Enjalbert, J. , Fournier, E. , Delmotte, F. & Dutech, C. (2008) Population genetics of fungal diseases of plants. Parasite, 15, 449–454. 18814721 10.1051/parasite/2008153449

[mpp13036-bib-0045] Grünwald, N.J. , McDonald, B.A. & Milgroom, M.G. (2016) Population genomics of fungal and oomycete pathogens. Annual Review of Phytopathology, 54, 323–346. 10.1146/annurev-phyto-080614-115913 27296138

[mpp13036-bib-0046] Hartl, D.L. & Clark, A.G. (1997) Principles of Population Genetics, 3rd edition. Sinauer Associates Inc.

[mpp13036-bib-0047] Hartman, G.L. , Bowen, C.R. , Haudenshield, J.S. , Fox, C.M. , Cary, T.R. & Diers, B.W. (2015) Evaluation of disease and pest damage on soybean cultivars released from 1923 through 2008 under field conditions in central Illinois. Agronomy Journal, 107, 2373–2380.

[mpp13036-bib-0048] Hartman, G.L. , Manandhar, J.B. & Sinclair, J.B. (1986) Incidence of *Colletotrichum* spp. on soybean and weeds in Illinois and pathogenicity of *Colletotrichum truncatum* . Phytopathology, 70, 780–782.

[mpp13036-bib-0049] Hartman, G.L. , Sinclair, J.B. & Rupe, J.C. (1999) Compendium of soybean diseasses, 4th edition. APS Press.

[mpp13036-bib-0050] Hartman, G.L. , West, E.D. & Herman, T.K. (2011) Crops that feed the world 2. Soybean—worldwide production, use, and constraints caused by pathogens and pests. Food Security, 3, 5–17.

[mpp13036-bib-0051] Helyar, S.J. , Hemmer‐Hansen, J. , Bekkevold, D. , Taylor, M.I. , Ogden, R. , Limborg, M.T. et al. (2011) Application of SNPs for population genetics of nonmodel organisms: New opportunities and challenges: Analytical approaches. Molecular Ecology Resources, 11, 123–136. 21429169 10.1111/j.1755-0998.2010.02943.x

[mpp13036-bib-0052] Huelsenbeck, J.P. & Ronquist, F. (2001) MRBAYES: Bayesian inference of phylogenetic trees. Bioinformatics, 17, 754–755. 11524383 10.1093/bioinformatics/17.8.754

[mpp13036-bib-0053] Jayawardena, R.S. , Hyde, K.D. , Damm, U. , Cai, L. , Liu, M. , Li, X.H. , Zhang, W. , Zhao, W.S. & Yan, J.Y. (2016) Notes on currently accepted species of *Colletotrichum* . Mycosphere, 7, 1192–1260.

[mpp13036-bib-0054] Kale, S.L. & Barhate, B.G. (2016) Management of anthracnose in soybean caused by *Colletotrichum truncatum* . International Journal of Plant Protection, 9, 583–588.

[mpp13036-bib-0055] Katoch, A. , Sharma, P. , Padder, B.A. & Sharma, P.N. (2017) Population structure of *Colletotrichum truncatum* in Himachal Pradesh and identification of broad‐spectrum resistant sources in *Capsicum* . Agricultural Research, 6, 296–303.

[mpp13036-bib-0056] Katoh, K. (2002) MAFFT: A novel method for rapid multiple sequence alignment based on fast Fourier transform. Nucleic Acids Research, 30, 3059–3066. 12136088 10.1093/nar/gkf436 PMC135756

[mpp13036-bib-0057] Katoh, K. & Standley, D.M. (2013) MAFFT multiple sequence alignment software version 7: Improvements in performance and usability. Molecular Biology and Evolution, 30, 772–780. 23329690 10.1093/molbev/mst010 PMC3603318

[mpp13036-bib-0058] Khan, M. & Sinclair, J.B. (1991) Effect of soil temperature on infection of soybean roots by sclerotia‐forming isolates of *Colletotrichum truncatum* . Plant Disease, 75, 1282–1285.

[mpp13036-bib-0059] Klosterman, S.J. , Rollins, J.R. , Sudarshana, M.R. & Vinatzer, B.A. (2016) Disease management in the genomics era—summaries of focus issue papers. Phytopathology, 106, 1068–1070. 27482626 10.1094/PHYTO-07-16-0276-FI

[mpp13036-bib-0060] Kumar, R. , Gupta, A. , Srivastava, S. , Devi, G. , Singh, V.K. , Goswami, S.K. et al. (2020) Diagnosis and detection of seed‐borne fungal phytopathogens. In: Kumar, R. and Gupta, A. (Eds.) Seed‐Borne Diseases of Agricultural Crops: Detection, Diagnosis and Management. : Springer Singapore, pp. 107–142.

[mpp13036-bib-0061] Lehman, S.G. & Wolf, F.A. (1926) Soybean anthracnose. Journal of Agricultural Research, 33, 381–390.

[mpp13036-bib-0062] Leite, T.S. , Cnossen‐Fassoni, A. , Pereira, O.L. , Mizubuti, E.S.G. , Araújo, E.F. & Queiroz, M.V. (2013) Novel and highly diverse fungal endophytes in soybean revealed by the consortium of two different techniques. Journal of Microbiology, 51, 56–69. 10.1007/s12275-013-2356-x 23456713

[mpp13036-bib-0063] Lenman, M. , Ali, A. , Mühlenbock, P. , Carlson‐Nilsson, U. , Liljeroth, E. , Champouret, N. et al. (2016) Effector‐driven marker development and cloning of resistance genes against *Phytophthora infestans* in potato breeding clone SW93‐1015. Theoretical and Applied Genetics, 129, 105–115. 26518573 10.1007/s00122-015-2613-y

[mpp13036-bib-0064] Liu, F. , Cai, L. , Crous, P.W. & Damm, U. (2014) The *Colletotrichum gigasporum* species complex. Persoonia, 33, 83–97. 25737595 10.3767/003158514X684447 PMC4312939

[mpp13036-bib-0065] Liu, F. , Wang, M. , Damm, U. , Crous, P.W. & Cai, L. (2016) Species boundaries in plant pathogenic fungi: A *Colletotrichum* case study. BMC Evolutionary Biology, 16, 81. 27080690 10.1186/s12862-016-0649-5 PMC4832473

[mpp13036-bib-0066] Liu, F. , Weir, B.S. , Damm, U. , Crous, P.W. , Wang, Y. , Liu, B. et al. (2015) Unravelling *Colletotrichum* species associated with *Camellia*: Employing *ApMat* and *GS* loci to resolve species in the *C. gloeosporioides* complex. Persoonia, 35, 63–86. 26823629 10.3767/003158515X687597 PMC4713112

[mpp13036-bib-0067] Luikart, G. , England, P.R. , Tallmon, D. , Jordan, S. & Taberlet, P. (2003) The power and promise of population genomics: From genotyping to genome typing. Nature Reviews Genetics, 4, 981–994. 10.1038/nrg1226 14631358

[mpp13036-bib-0068] Madden, L.V. (1997) Effects of rain on splash dispersal of fungal pathogens. Canadian Journal of Plant Pathology, 19, 225–230.

[mpp13036-bib-0069] Mahmodi, F. , Kadir, J.B. , Wong, M.Y. , Nasehi, A. , Puteh, A. & Soleimani, N. (2013) First report of anthracnose caused by *Colletotrichum gloeosporioides* on soybean (*Glycine max*) in Malaysia. Plant Disease, 97, 841. 10.1094/PDIS-10-12-0944-PDN 30722625

[mpp13036-bib-0070] Manandhar, J.B. , Hartman, G.L. & Sinclair, J.B. (1986) *Colletotrichum destructivum*, the anamorph of *Glomerella glycines* . Phytopathology, 76, 282–285.

[mpp13036-bib-0071] Manandhar, J.B. , Kunwar, K. , Tribhuwan Singh, G.L. , Hartman, G.L. & Sinclair, J.B. (1985) Penetration and infection of soybean leaf tissues by *Colletotrichum truncatum* and *Glomerella glycine* . Phytopathology, 75, 704–708.

[mpp13036-bib-0072] Mancini, V. , Murolo, S. & Romanazzi, G. (2016) Diagnostic methods for detecting fungal pathogens on vegetable seeds. Plant Pathology, 65, 691–703.

[mpp13036-bib-0073] Marin‐Felix, Y. , Groenewald, J.Z. , Cai, L. , Chen, Q. , Marincowitz, S. , Barnes, I. et al. (2017) Genera of phytopathogenic fungi: GOPHY 1. Studies in Mycology, 86, 99–216. 28663602 10.1016/j.simyco.2017.04.002 PMC5486355

[mpp13036-bib-0074] McDonald, B.A. (1997) The population genetics of fungi: Tools and techniques. Phytopathology, 87, 448–453. 18945126 10.1094/PHYTO.1997.87.4.448

[mpp13036-bib-0075] McDonald, B.A. & Linde, C. (2002) Pathogen population genetics, evolutionary potential, and durable resistance. Annual Review of Phytopathology, 40, 349–379. 10.1146/annurev.phyto.40.120501.101443 12147764

[mpp13036-bib-0076] Nagaraj, B.T. , Jahagirdar, S. & Basavaraja, G.T. (2014) Identification of resistant sources in glass house and field evaluation of soybean genotypes to anthracnose caused by *Colletotrichum truncatum* (Schew.) Andrus and Moore. The Bioscan, 9, 1333–1336.

[mpp13036-bib-0077] Nakata, K. & Takimoto, K. (1934) A list of crop diseases in Korea. In Agricultural experiment station governmental central chosen research report, 15, 1–146.

[mpp13036-bib-0078] Nataraj, V. , Maranna, S. , Kumawat, G. , Gupta, S. , Rajput, L.S. , Kumar, S. et al. (2020) Genetic inheritance and identification of germplasm sources for anthracnose resistance in soybean [*Glycine max* (L.) Merr.]. Genetic Resources and Crop Evolution, 67, 1449–1456.

[mpp13036-bib-0079] Nilsson, R.H. , Ryberg, M. , Kristiansson, E. , Abarenkov, K. , Larsson, K.‐H. & Kõljalg, U. (2006) Taxonomic reliability of DNA sequences in public sequence databases: A fungal perspective. PLoS One, 1, e59. 17183689 10.1371/journal.pone.0000059 PMC1762357

[mpp13036-bib-0080] Notomi, T. , Okayama, H. , Masubuchi, H. , Yonekawa, T. , Watanabe, K. , Amino, N. & Hase, T. (2000) Loop‐mediated isothermal amplification of DNA. Nucleic Acids Research, 28, e63. 10.1093/nar/28.12.e63 PMC102748 10871386

[mpp13036-bib-0081] O'Connell, R.J. , Thon, M.R. , Hacquard, S. , Amyotte, S.G. , Kleemann, J. , Torres, M.F. et al. (2012) Lifestyle transitions in plant pathogenic *Colletotrichum* fungi deciphered by genome and transcriptome analyses. Nature Genetics, 44, 1060–1065. 22885923 10.1038/ng.2372 PMC9754331

[mpp13036-bib-0082] Oliver, R.P. & Hewitt, H.G. (2014) Fungicides in Crop Protection. Wallingford, UK: CABI.

[mpp13036-bib-0083] Oliver, R.P. & Solomon, P.S. (2010) New developments in pathogenicity and virulence of necrotrophs. Current Opinion in Plant Biology, 13, 415–419. 20684067

[mpp13036-bib-0084] Pagano, M.C. & Miransari, M. (2016) The importance of soybean production worldwide. In: Miransari, M. (Ed.) Abiotic and Biotic Stresses in Soybean Production. Cambridge, MA, USA: Academic Press, pp. 1–26.

[mpp13036-bib-0085] Pecchia, S. , Caggiano, B. , Da Lio, D. , Cafà, G. , Le Floch, G. & Baroncelli, R. (2019) Molecular detection of the seed‐borne pathogen *Colletotrichum lupini* targeting the hyper‐variable IGS region of the ribosomal cluster. Plants, 8, 222. 10.3390/plants8070222 PMC6681257 31337095

[mpp13036-bib-0086] Pellegrino, C. , Gilardi, G. , Gullino, M.L. & Garibaldi, A. (2010) Detection of *Phoma valerianellae* in lambs lettuce seeds. Phytoparasitica, 38, 159–165.

[mpp13036-bib-0087] Perfect, S.E. , Hughes, H.B. , O’Connell, R.J. & Green, J.R. (1999) *Colletotrichum*: A model genus for studies on pathology and fungal–plant interactions. Fungal Genetics and Biology, 27, 186–198. 10441444 10.1006/fgbi.1999.1143

[mpp13036-bib-0088] Pieck, M.L. , Ruck, A. , Farman, M.L. , Peterson, G.L. , Stack, J.P. , Valent, B. et al. (2017) Genomics‐based marker discovery and diagnostic assay development for wheat blast. Plant Disease, 101, 103–109. 30682315 10.1094/PDIS-04-16-0500-RE

[mpp13036-bib-0089] Plissonneau, C. , Benevenuto, J. , Mohd‐Assaad, N. , Fouché, S. , Hartmann, F.E. & Croll, D. (2017) Using population and comparative genomics to understand the genetic basis of effector‐driven fungal pathogen evolution. Frontiers in Plant Science, 8, 119. 28217138 10.3389/fpls.2017.00119 PMC5289978

[mpp13036-bib-0090] Poti, T. , Mahawan, K. , Cheewangkoon, R. , Arunothayanan, H. , Akimitsu, K. & Nalumpang, S. (2020) Detection and molecular characterization of carbendazim‐resistant *Colletotrichum truncatum* isolates causing anthracnose of soybean in Thailand. Journal of Phytopathology, 168, 267–278.

[mpp13036-bib-0091] Prasad, P. , Savadi, S. , Bhardwaj, S.C. , Gangwar, O.P. & Kumar, S. (2019) Rust pathogen effectors: Perspectives in resistance breeding. Planta, 250, 1–22. 30980247 10.1007/s00425-019-03167-6

[mpp13036-bib-0092] Prusky, D. (1996) Pathogen quiescene in postharvest diseases. Annual Review of Phytopathology, 34, 413–434. 10.1146/annurev.phyto.34.1.413 15012550

[mpp13036-bib-0093] Prusky, D. , Alkan, N. , Mengiste, T. & Fluhr, R. (2013) Quiescent and necrotrophic lifestyle choice during postharvest disease development. Annual Review of Phytopathology, 51, 155–176. 10.1146/annurev-phyto-082712-102349 23682917

[mpp13036-bib-0094] Raffaele, S. & Kamoun, S. (2012) Genome evolution in filamentous plant pathogens: Why bigger can be better. Nature Reviews Microbiology, 10, 417–430. 22565130 10.1038/nrmicro2790

[mpp13036-bib-0095] Rambaut, R. (2014) FigTree vol 1.4.2, a graphical viewer of phylogenetic trees. Available at: http://tree.bio.ed.ac.uk/software/figtree/ [Accessed 8 January 2021].

[mpp13036-bib-0096] Ramiro, J. , Ciampi‐Guillardi, M. , Caldas, D.G.G. , de Moraes, M.H.D. , Barbieri, M.C.G. , Pereira, W.V. et al. (2019) Quick and accurate detection of *Sclerotinia sclerotiorum* and *Phomopsis* spp. in soybean seeds using qPCR and seed‐soaking method. Journal of Phytopathology, 167, 273–282.

[mpp13036-bib-0097] Ramos, A.M. , Gally, M. , García, M.C. & Levin, L. (2010) Pectinolytic enzyme production by *Colletotrichum truncatum*, causal agent of soybean anthracnose. Revista Iberoamericana De Micología, 27, 186–190. 20627133 10.1016/j.riam.2010.06.002

[mpp13036-bib-0098] Ramos, A.M. , Tadic, L.F. , Cinto, I. , Carmona, M. & Gally, M. (2013) Molecular characterization of *Colletotrichum* species causing soybean anthracnose in Argentina. Mycotaxon, 123, 457–465.

[mpp13036-bib-0099] Ranathunge, N.P. , Ford, R. & Taylor, P.W.J. (2009) Development and optimization of sequence‐tagged microsatellite site markers to detect genetic diversity within *Colletotrichum capsici*, a causal agent of chilli pepper anthracnose disease. Molecular Ecology Resources, 9, 1175–1179. 21564867 10.1111/j.1755-0998.2009.02608.x

[mpp13036-bib-0100] Rao, S. & Nandineni, M.R. (2017) Genome sequencing and comparative genomics reveal a repertoire of putative pathogenicity genes in chilli anthracnose fungus *Colletotrichum truncatum* . PLoS One, 12, e0183567. 28846714 10.1371/journal.pone.0183567 PMC5573122

[mpp13036-bib-0101] Riccioni, L. , Conca, G. & Hartman, G.L. (1998) First report of *Colletotrichum coccodes* on soybean in the United States. Plant Disease, 82, 959. 10.1094/PDIS.1998.82.8.959C 30856932

[mpp13036-bib-0102] Rogério, F. , Boufleur, T.R. , Ciampi‐Guillardi, M. , Sukno, S.A. , Thon, M.R. , Massola Júnior, N.S. , et al. (2020) Genome sequence resources of *Colletotrichum truncatum*. *C. plurivorum*, *C. musicola*, and *C. sojae*: Four species pathogenic to soybean (*Glycine max*). Phytopathology, 110, 1497–1499. 32352862 10.1094/PHYTO-03-20-0102-A

[mpp13036-bib-0103] Rogério, F. , Ciampi‐Guillardi, M. , Barbieri, M.C.G. , Bragança, C.A.D. , Seixas, C.D.S. , Almeida, A.M.R. et al. (2017) Phylogeny and variability of *Colletotrichum truncatum* associated with soybean anthracnose in Brazil. Journal of Applied Microbiology, 122, 402–415. 27859958 10.1111/jam.13346

[mpp13036-bib-0104] Rogério, F. , Gladieux, P. , Massola, N.S. & Ciampi‐Guillardi, M. (2019) Multiple introductions without admixture of *Colletotrichum truncatum* associated with soybean anthracnose in Brazil. Phytopathology, 109, 681–689. 30451637 10.1094/PHYTO-08-18-0321-R

[mpp13036-bib-0105] Sant’Anna, J.R. , Miyamoto, C.T. , Rosada, L.J. , Franco, C.C.S. , Kaneshima, E.N. & Castro‐Prado, M.A.A. (2010) Genetic relatedness of Brazilian *Colletotrichum truncatum* isolates assessed by vegetative compatibility groups and RAPD analysis. Biological Research, 43, 51–62. 21157632

[mpp13036-bib-0106] Sarrocco, S. , Herrera‐Estrella, A. & Collinge, D.B. (2020) Editorial: Plant disease management in the post‐genomic era: From functional genomics to genome editing. Frontiers in Microbiology, 11, 107. 32117135 10.3389/fmicb.2020.00107 PMC7010928

[mpp13036-bib-0107] Schena, L. , Abdelfattah, A. , Mosca, S. , Li Destri Nicosia, M.G. , Agosteo, G.E. & Cacciola, S.O. (2017) Quantitative detection of *Colletotrichum godetiae* and *C. acutatum* sensu stricto in the phyllosphere and carposphere of olive during four phenological phases. European Journal of Plant Pathology, 149, 337–347.

[mpp13036-bib-0108] Sharma, R. (2009) Genetic differentiation of host limited forms of *Colletotrichum truncatum* from northwestern Himalayas. Archives of Phytopathology and Plant Protection, 42, 960–966.

[mpp13036-bib-0109] Sharma, S.K. , Gupta, G.K. & Ramteke, R. (2011) *Colletotrichum truncatum* [(Schw.) Andrus and W.D. Moore], the causal agent of anthracnose of soybean [*Glycine max* (L.) Merrill.] – a review. Soybean Research, 9, 31–52.

[mpp13036-bib-0110] Shi, X. , Wang, S. , Duan, X. , Gao, X. , Zhu, X. & Laborda, P. (2020) First report of *Colletotrichum brevisporum* causing soybean antrhacnose in China. Plant Disease, 10.1094/PDIS-09-20-1910-PDN

[mpp13036-bib-0035] de Silva, D.D. , Crous, P.W. , Ades, P.K. , Hyde, K.D. & Taylor, P.W.J. (2017) Lifestyles of *Colletotrichum* species and implications for plant biosecurity. Fungal Biology Reviews, 31, 155–168.

[mpp13036-bib-0029] da Silva, L.L. , Moreno, H.L.A. , Correia, H.L.N. , Santana, M.F. & de Queiroz, M.V. (2020) *Colletotrichum*: Species complexes, lifestyle, and peculiarities of some sources of genetic variability. Applied Microbiology and Biotechnology, 104, 1891–1904. 31932894 10.1007/s00253-020-10363-y

[mpp13036-bib-0136] Sinclair, J.B. (1989) Compendium of Soybean Diseases, 3rd edition. St Paul, MN, USA: American Phytopathological Society.

[mpp13036-bib-0112] Stecher, G. , Tamura, K. & Kumar, S. (2020) Molecular evolutionary genetics analysis (MEGA) for macOS. Molecular Biology and Evolution, 37, 1237–1239. 31904846 10.1093/molbev/msz312 PMC7086165

[mpp13036-bib-0113] Stukenbrock, E.H. (2016) The role of hybridization in the evolution and emergence of new fungal plant pathogens. Phytopathology, 106, 104–112. 26824768 10.1094/PHYTO-08-15-0184-RVW

[mpp13036-bib-0114] Stukenbrock, E.H. & Bataillon, T. (2012) A population genomics perspective on the emergence and adaptation of new plant pathogens in agro‐ecosystems. PLoS Pathogens, 8, e1002893. 23028308 10.1371/journal.ppat.1002893 PMC3460620

[mpp13036-bib-0115] Subedi, S. , Gharti, D.B. , Neupane, S. & Ghimire, T. (2016) Management of anthracnose in soybean using fungicide. Journal of Nepal Agricultural Research Council, 1, 29–32.

[mpp13036-bib-0116] Talhinhas, P. , Baroncelli, R. & Le Floch, G. (2016) Anthracnose of lupins caused by *Colletotrichum lupini*: A recent disease and a successful worldwide pathogen. Journal of Plant Pathology, 98, 5–14.

[mpp13036-bib-0117] Thierry, M. , Gladieux, P. , Fournier, E. , Tharreau, D. & Ioos, R. (2020) A genomic approach to develop a new qPCR test enabling detection of the *Pyricularia oryzae* lineage causing wheat blast. Plant Disease, 104, 60–70. 31647693 10.1094/PDIS-04-19-0685-RE

[mpp13036-bib-0118] Tian, Q. , Lu, C. , Wang, S. , Xiong, Q. , Zhang, H. , Wang, Y. & et al (2017) Rapid diagnosis of soybean anthracnose caused by *Colletotrichum truncatum* using a loop‐mediated isothermal amplification (LAMP) assay. European Journal of Plant Pathology, 148, 785–793.

[mpp13036-bib-0119] Ureña‐Padilla, A.R. , MacKenzie, S.J. , Bowen, B.W. & Legard, D.E. (2002) Etiology and population genetics of *Colletotrichum* spp. causing crown and fruit rot of strawberry. Phytopathology, 92, 1245–1252. 18944251 10.1094/PHYTO.2002.92.11.1245

[mpp13036-bib-0120] USDA (2020) World Agricultural Production. Circular Series WAP 12‐20. USA: USDA. https://downloads.usda.library.cornell.edu/usda‐esmis/files/5q47rn72z/cr56ns297/00000r529/production.pdf

[mpp13036-bib-0121] Van de Wouw, A.P. & Idnurm, A. (2019) Biotechnological potential of engineering pathogen effector proteins for use in plant disease management. Biotechnology Advances, 37, 107387. 31022532 10.1016/j.biotechadv.2019.04.009

[mpp13036-bib-0122] Vasconcelos, M.J.V. , Machado, M.A. , Almeida, A.M.D. , Henning, A.A. , de Barros, E.G. & Moreira, M.A. (1994) Differentiation of *Colletotrichum truncatum* isolates by random amplified polymorphic DNA. Fitopatologia Brasileira, 19, 520–523.

[mpp13036-bib-0123] Vieira, W.A.D.S. , Bezerra, P.A. , Silva, A.C.D. , Veloso, J.S. , Câmara, M.P.S. & Doyle, V.P. (2020) Optimal markers for the identification of *Colletotrichum* species. Molecular Phylogenetics and Evolution, 143, 106694. 31786239 10.1016/j.ympev.2019.106694

[mpp13036-bib-0124] Wang, S. , Ye, W. , Tian, Q. , Dong, S. & Zheng, X. (2017) Rapid detection of *Colletotrichum gloeosporioides* using a loop‐mediated isothermal amplification assay. Australasian Plant Pathology, 46, 493–498.

[mpp13036-bib-0125] Weir, B.S. , Johnston, P.R. & Damm, U. (2012) The *Colletotrichum gloeosporioides* species complex. Studies in Mycology, 73, 115–180. 23136459 10.3114/sim0011 PMC3458417

[mpp13036-bib-0126] Wrather, A. , Shannon, G. , Balardin, R. , Carregal, L. , Escobar, R. , Gupta, G.K. et al. (2010) Effect of diseases on soybean yield in the top eight producing countries in 2006. Plant Health Progress, 11, 29.

[mpp13036-bib-0127] Xavier, K.V. , Mizubuti, E.S.G. , Queiroz, M.V. , Chopra, S. & Vaillancourt, L. (2018) Genotypic and pathogenic diversity of *Colletotrichum sublineola* isolates from sorghum (*Sorghum bicolor*) and johnsongrass (*S. halepense*) in the southeastern United States. Plant Disease, 102, 2341–2351. 30199327 10.1094/PDIS-04-18-0562-RE

[mpp13036-bib-0128] Yang, H.‐C. & Hartman, G.L. (2015) Methods and evaluation of soybean genotypes for resistance to *Colletotrichum truncatum* . Plant Disease, 99, 143–148. 30699740 10.1094/PDIS-03-14-0228-RE

[mpp13036-bib-0129] Yang, H.C. & Hartman, G.L. (2016) Anthracnose. In: Hartman, G.L. , Rupe, J. and Sikora, E.J. (Eds.) Compendium of Soybean Diseases and Pests. St Paul, MN, USA: American Phytopathological Society, pp. 31–34.

[mpp13036-bib-0130] Yang, H.‐C. , Haudenshield, J.S. & Hartman, G.L. (2012) First report of *Colletotrichum chlorophyti* causing soybean anthracnose. Plant Disease, 96, 1699. 10.1094/PDIS-06-12-0531-PDN 30727470

[mpp13036-bib-0131] Yang, H.‐C. , Haudenshield, J.S. & Hartman, G.L. (2014) *Colletotrichum incanum* sp. nov., a curved‐conidial species causing soybean anthracnose in USA. Mycologia, 106, 32–42. 24603833 10.3852/13-013

[mpp13036-bib-0132] Yang, H.‐C. , Haudenshield, J.S. & Hartman, G.L. (2015) Multiplex real‐time PCR detection and differentiation of *Colletotrichum* species infecting soybean. Plant Disease, 99, 1559–1568. 30695948 10.1094/PDIS-11-14-1189-RE

[mpp13036-bib-0133] Yang, H.‐C. , Stewart, J.M. & Hartman, G.L. (2013) First report of *Colletotrichum chlorophyti* infecting soybean seed in Arkansas, United States. Plant Disease, 97, 1510. 10.1094/PDIS-04-13-0441-PDN 30708473

[mpp13036-bib-0134] Zaw, M. , Aye, S.S. & Matsumoto, M. (2020) *Colletotrichum* and *Diaporthe* species associated with soybean stem diseases in Myanmar. Journal of General Plant Pathology, 86, 114–123.

[mpp13036-bib-0135] Zhan, J. (2009) Population genetics of plant pathogens. In: Encyclopedia of Life Sciences. John Wiley and Sons Ltd, a0021269. https://onlinelibrary.wiley.com/doi/10.1002/9780470015902.a0021269

